# *GhIMP10D,* an inositol monophosphates family gene, enhances ascorbic acid and antioxidant enzyme activities to confer alkaline tolerance in *Gossypium hirsutum* L.

**DOI:** 10.1186/s12870-023-04462-x

**Published:** 2023-09-22

**Authors:** Yapeng Fan, Fanjia Peng, Ruifeng Cui, Shuai Wang, Yupeng Cui, Xuke Lu, Hui Huang, Kesong Ni, Xiaoyu Liu, Tiantian Jiang, Xixian Feng, Mengyue Liu, Yuqian Lei, Wenhua Chen, Yuan Meng, Mingge Han, Delong Wang, Zujun Yin, Xiugui Chen, Junjuan Wang, Yujun Li, Lixue Guo, Lanjie Zhao, Wuwei Ye

**Affiliations:** 1 Institute of Cotton Research of Chinese Academy of Agricultural Sciences / Research Base, National Key Laboratory of Cotton Bio-Breeding and Integrated Utilization, Anyang Institute of Technology, Henan, 455000 China; 2 Hunan Institute of Cotton Science, Hunan, 415101 China

**Keywords:** Inositol monophosphates, Ascorbic acid, Alkaline tolerance, *Gossypium hirsutum* L.

## Abstract

**Background:**

Inositol monophosphates (IMP) are key enzymes in the ascorbic acid (AsA) synthesis pathways, which play vital roles in regulating plant growth and development and stresses tolerance. To date, no comprehensive analysis of the expression profile of *IMP* genes and their functions under abiotic stress in cotton has been reported.

**Results:**

In this study, the genetic characteristics, phylogenetic evolution, *cis*-acting elements and expression patterns of *IMP* gene family in cotton were systematically analyzed. A total of 28, 27, 13 and 13 *IMP* genes were identified in *Gossypium hirsutum* (*G. hirsutum*), *Gossypium barbadense* (*G. barbadense*), *Gossypium arboreum* (*G. arboreum*), and *Gossypium raimondii* (*G. raimondii*), respectively. Phylogenetic analysis showed that *IMP* family genes could cluster into 3 clades. Structure analysis of genes showed that *GhIMP* genes from the same subgroup had similar genetic structure and exon number. And most *GhIMP* family members contained hormone-related elements (abscisic acid response element, MeJA response element, gibberellin response element) and stress-related elements (low temperature response element, defense and stress response element, wound response element). After exogenous application of abscisic acid (ABA), some *GhIMP* genes containing ABA response elements positively responded to alkaline stress, indicating that ABA response elements played an important role in response to alkaline stress. qRT-PCR showed that most of *GhIMP* genes responded positively to alkaline stress, and *GhIMP10D* significantly upregulated under alkaline stress, with the highest up-regulated expression level. Virus-induced gene silencing (VIGS) experiment showed that compared with 156 plants, MDA content of pYL156:*GhIMP10D* plants increased significantly, while POD, SOD, chlorophyII and AsA content decreased significantly.

**Conclusions:**

This study provides a thorough overview of the *IMP* gene family and presents a new perspective on the evolution of this gene family. In particular, some *IMP* genes may be involved in alkaline stress tolerance regulation, and *GhIMP10D* showed high expression levels in leaves, stems and roots under alkaline stress, and preliminary functional verification of *GhIMP10D* gene suggested that it may regulate tolerance to alkaline stress by regulating the activity of antioxidant enzymes and the content of AsA. This study contributes to the subsequent broader discussion of the structure and alkaline resistance of *IMP* genes in cotton.

**Supplementary Information:**

The online version contains supplementary material available at 10.1186/s12870-023-04462-x.

## Background

Saline-alkaline stress seriously inhibits crops growth and yield, and it was reported that over 954 million hectares of soils in the world were poisoned by saline-alkaline conditions [1]. Soil salinity is often accompanied by alkalinity because the soil tends to contain sodium carbonate (Na_2_CO_3_) and sodium bicarbonate (NaHCO_3_), raising the pH of the soil. Alkaline stress can cause osmotic stress and ion damage like salt stress [2], but with the additional influence of high pH. High pH environment at the rhizosphere can precipitate Fe^2+^, Mn^2+^, Ca^2+^, Mg^2+^ and HPO_3_^−^, thus reducing the availability of mineral elements, inhibiting ion absorption, and destroying ion homeostasis [3]. It was found that reactive oxygen species (ROS) and malondialdehyde (MDA) would accumulate in large quantities under alkaline stress, which in turn destroyed cell membrane structure and intracellular components [4]. Therefore, excessive ROS will accumulate under stresses, causing a serious impact on plant growth and development [5].

Although alkaline stress is serious, plants also develop a series of measures to resist stresses. To eliminate harmful ROS produced by stress, plants have evolved enzymatic and non-enzymatic systems to cope with abiotic stress. The enzyme system is mainly composed of superoxide dismutase (SOD), catalase (CAT), guaiacol peroxidase (GPX), glutathione peroxidase (GSH-Px) and ascorbate peroxidase (APX). And reduced glutathione (GSH) and AsA are the main components of the non-enzymatic system. As an antioxidant molecule in the non-enzymatic system, AsA is a key substrate in the elimination of ROS [6–8]. AsA can widely participate in the response to adversity stresses, such as salt stress [9, 10] and drought stress [11], by regulating antioxidant defense system and activating different antioxidant enzymes. According to our previous study [12], genes in the ascorbate synthesis pathway were differentially expressed under alkaline stress, and *IMP* genes played a key role in the synthesis of ascorbic acid.

Phosphatases in the inositol signaling pathway can be classified into the following categories: inositol polyphosphate 5-phosphatases (5PTases), suppressor of actin (SAC) phosphatases, SAL1 phosphatase/FIERY1 (FRY1) and its homologs, inositol monophosphates (*IMP*), and phosphatase and tensin homologue deleted on chromosome 10 (PTEN)-related phosphatases [13]. It was also found that IMPase also catalyzed the dephosphorylation of L-galactose 1-phosphate and was a bi-functional enzyme as it participated in the biosynthesis of AsA [14]. Therefore, the *IMP*-encoded enzyme may exhibit bifunctionality in plants.

However, with the deepening of research in recent years, more and more evidences have shown that *IMP* genes are involved in abiotic stress by controlling the synthesis of AsA. In tobacco, overexpression of the *OsIMP* gene enhanced the activities of antioxidant enzymes and thus enhanced tolerance to cold stress [15]. In *Cicer arietinum* L., plants that overexpressed the *CaIMP* gene had higher AsA levels than wild type, suggesting *CaIMP* could improve plant tolerance to stress through AsA metabolism pathways [16].

Cotton is an important cash crop, and it is also a pioneer crop in salt-alkaline land with a certain degree of salt-alkaline tolerance, so it is an important model crop for us to study saline-alkaline stress [17]. In recent years, genome sequencing including 2 diploids, *G. arboreum* [18], and *G. raimondii* [19], and 2 allotetraploid, *G. hirsutum* [20, 21] and *G. barbadense* [22, 23], has been completed, which provides strong support for our study of the evolution of the *IMP* gene family and the potential function of the *IMP* genes.

To obtain the evolutionary characteristics and potential function of *IMP* gene family in cotton, we characterized *IMP* gene family in *G. hirsutum*, *G. barbadense*, *G. arboreum*, and *G. raimondii*. Then we visualized the chromosomal distribution and collinearity of four cotton species, later, gene ontology annotations, gene structures, conserved motifs, *cis*-acting elements of promoters and expression patterns under different stresses in *G. hirsutum* were also analyzed. Then, a highly expressed gene *GhIMP10D* induced by alkaline stress was selected, and the gene was silenced using VIGS technology to study its function. The objective of this study was to explore the evolutionary relationship of the *IMP* gene family and its potential function in alkaline tolerance.

## Results

### Identification of *IMP* family genes

**Fig. 1:**
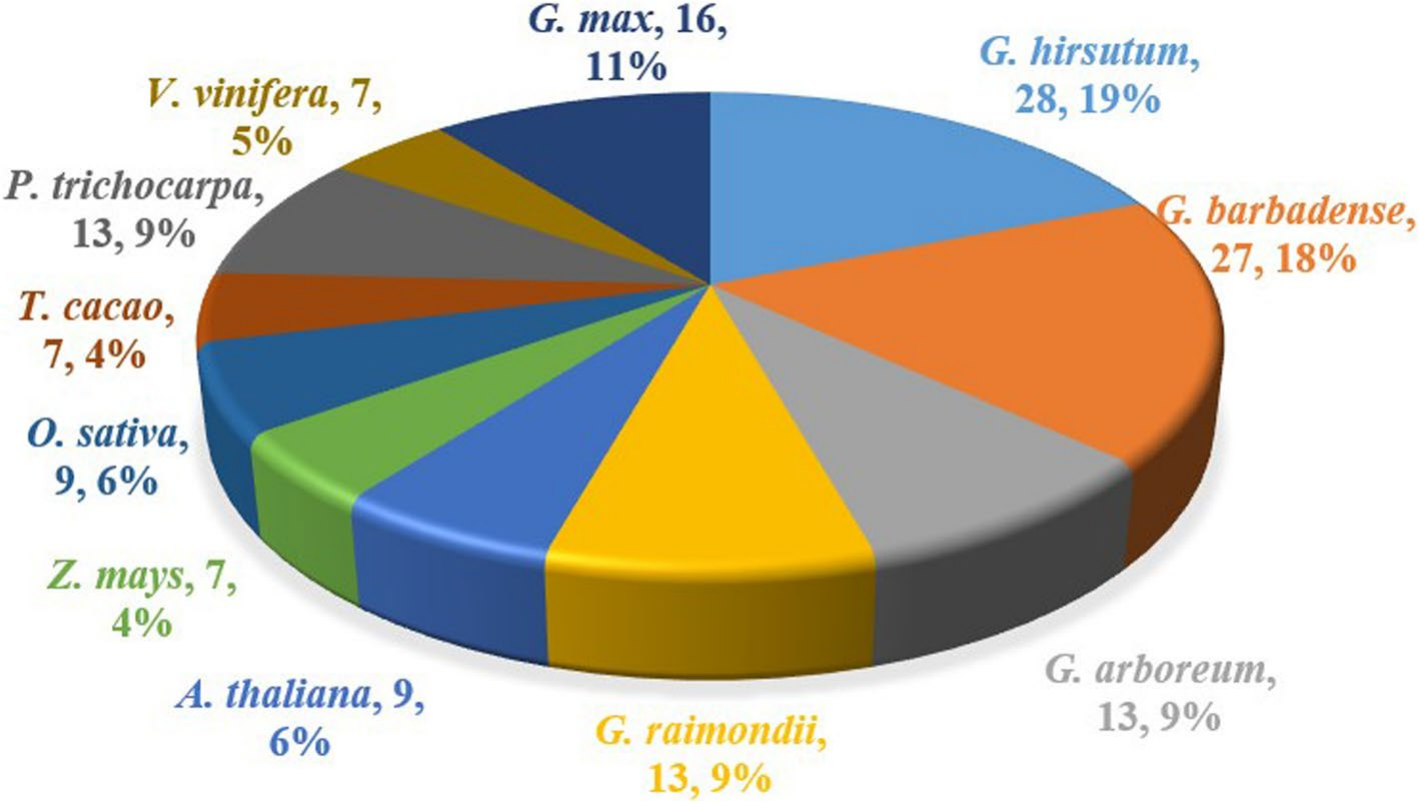
Distribution of *IMP* genes among four *Gossypium species* and other 7 species

To identify the members of the *IMP* gene family in four *Gossypium* species, a Hidden Markov Model (HMM) profile of inositol_P domain (PF00459) from Pfam (https://pfam.xfam.org/) as a query to search the *IMP* gene members in *G. hirsutum*, *G. barbadense, G. arboreum*, and *G. raimondii*. Then we used Pfam and NCBI-CD search to verify the *IMP* genes and deleted the genes with incomplete C and N terminals manually. Online website Phytozome v13 (https://phytozome-next.jgi.doe.gov/) was used to compare homologous genes of other species by using PF00459 as the keyword. Finally, a total of 28, 27, 13, 13, 9, 7, 9, 7, 13, 7 and 16 *IMP* genes were identified in *G. hirsutum*, *G. barbadense*, *G. arboreum*, and *G. raimondii*, *Arabidopsis thaliana* (*A. thaliana*), *Zea mays* (*Z. mays*), *Oryza sativa* (*O. sativa*), *Theobroma cacao* (*T. cacao*), *Populus trichocarpa* (*P. trichocarpa*), *Vitis vinifera* (*V. vinifera*) and *Glycine max* (*G. max*), respectively (Fig. 1). To better understand the gene arrangement of the *IMP* gene family and the differences in the number of genes among different species, we renamed the *IMP* genes according to their positions on the corresponding chromosomes. Specifically, the genes on *G. hirsutum* were renamed *GhIMP1A*-*GhIMP13D*, and the genes on *G. barbadense* were renamed *GbIMP1A*-*GbIMP13D*. Detailed results of gene renaming were shown in supplementary Table S1. The two tetraploid cotton species, *G. hirsutum* and *G. barbadense*, had twice the number of *IMP* family genes than the two diploid cotton species *G. arboreum* and *G. raimondii*. And the number of *IMP* genes in *G. hirsutum* and *G. barbadense* was higher than that of other species, indicating that the *IMP* gene family of this two cotton species expanded massively during the process of evolution.

### Phylogenetic analysis of *IMP* genes

To detect the evolutionary relationships of *IMP* proteins among four *Gossypium* species and other 7 species, MEGA 7 software was used to construct phylogenetic trees. It was worth noting that an unrooted phylogenetic tree between four *Gossypium* species was constructed based on the neighbor-joining (NJ) method with 1 000 bootstrap replicates, while a Maximum Likelihood (ML) tree was constructed between four *Gossypium* species and other 7 species.

**Fig. 2:**
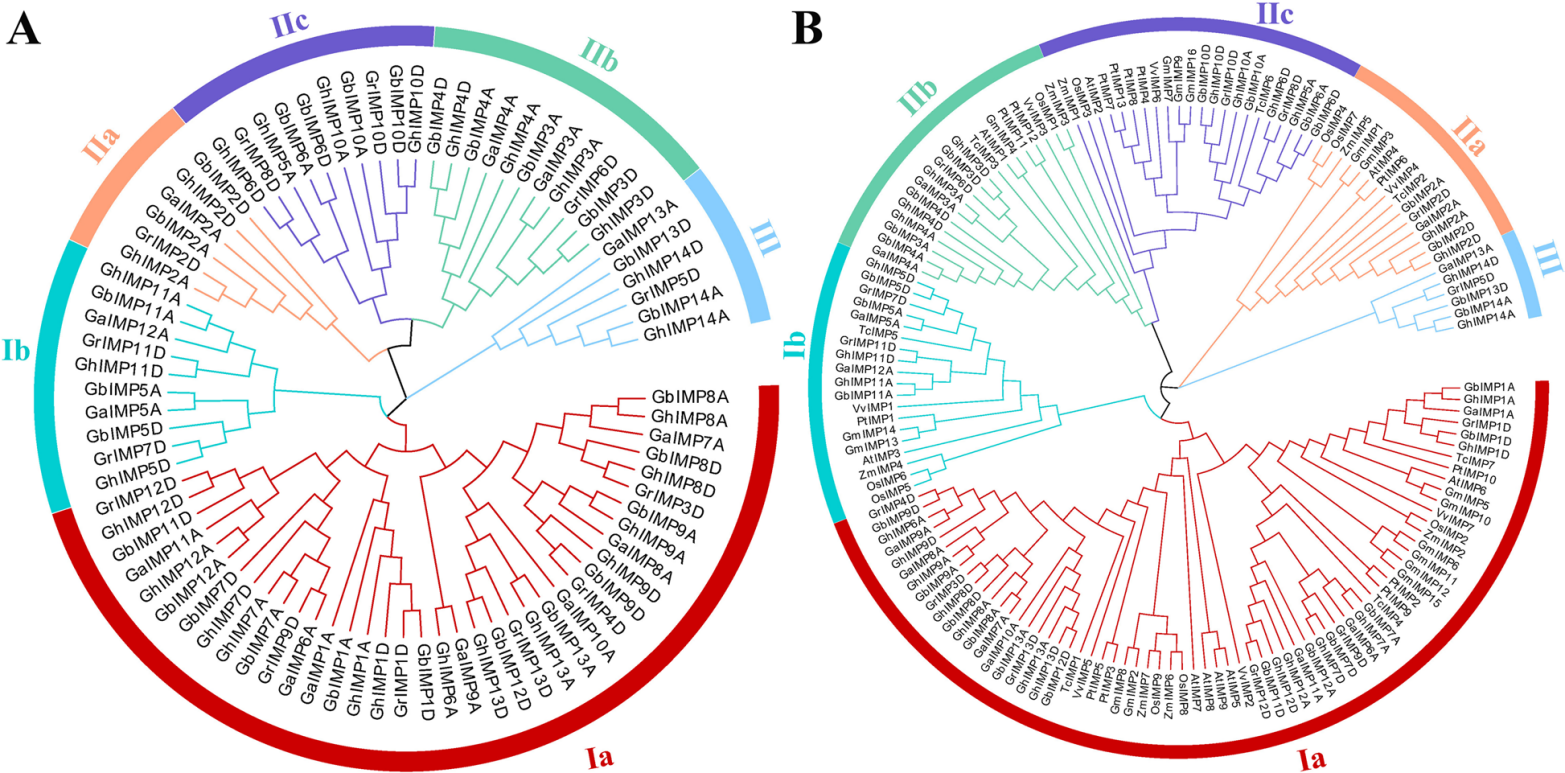
Phylogenetic trees of *IMP* genes in four *Gossypium* species and other 7 species. **A** Phylogenetic tree of the 81 *IMP* genes from four *Gossypium* species using MEGA 7 by the Neighbor-Joining (NJ) method. **B** Phylogenetic tree of the 149 *IMP* genes from four *Gossypium* species, *A. thaliana*, *Z. mays*, *O. sativa*, *T. cacao*, *P. trichocarpa*, *V. vinifera* and *G. max* using MEGA 7 by Maximum Likelihood (ML) method

According to the sequence similarity, tree topology and structural characteristics in each sequence, we divided the *IMP* genes into 3 clades, and each clade can be divided into different classes (Fig. 2). Among them, there were 2 classes in clade I with 87 genes, 3 in clade II with 56 genes, and 1 in clade III with 6 genes, and clade I had the most genes (Fig. 2B). The *IMP* genes in tetraploid cotton species were almost twice as many as diploid cotton species in each subgroup (Fig. 2A).

According to the analysis of evolutionary relationship, we can know that except clade III, the *IMP* genes of four cotton species in the other clades are more closely related to that of *T. cacao* than any other plant species, which is consistent with previous studies that cotton and cacao emerged from the same ancestor [21].

### Analysis of chromosomal localization

**Fig. 3:**
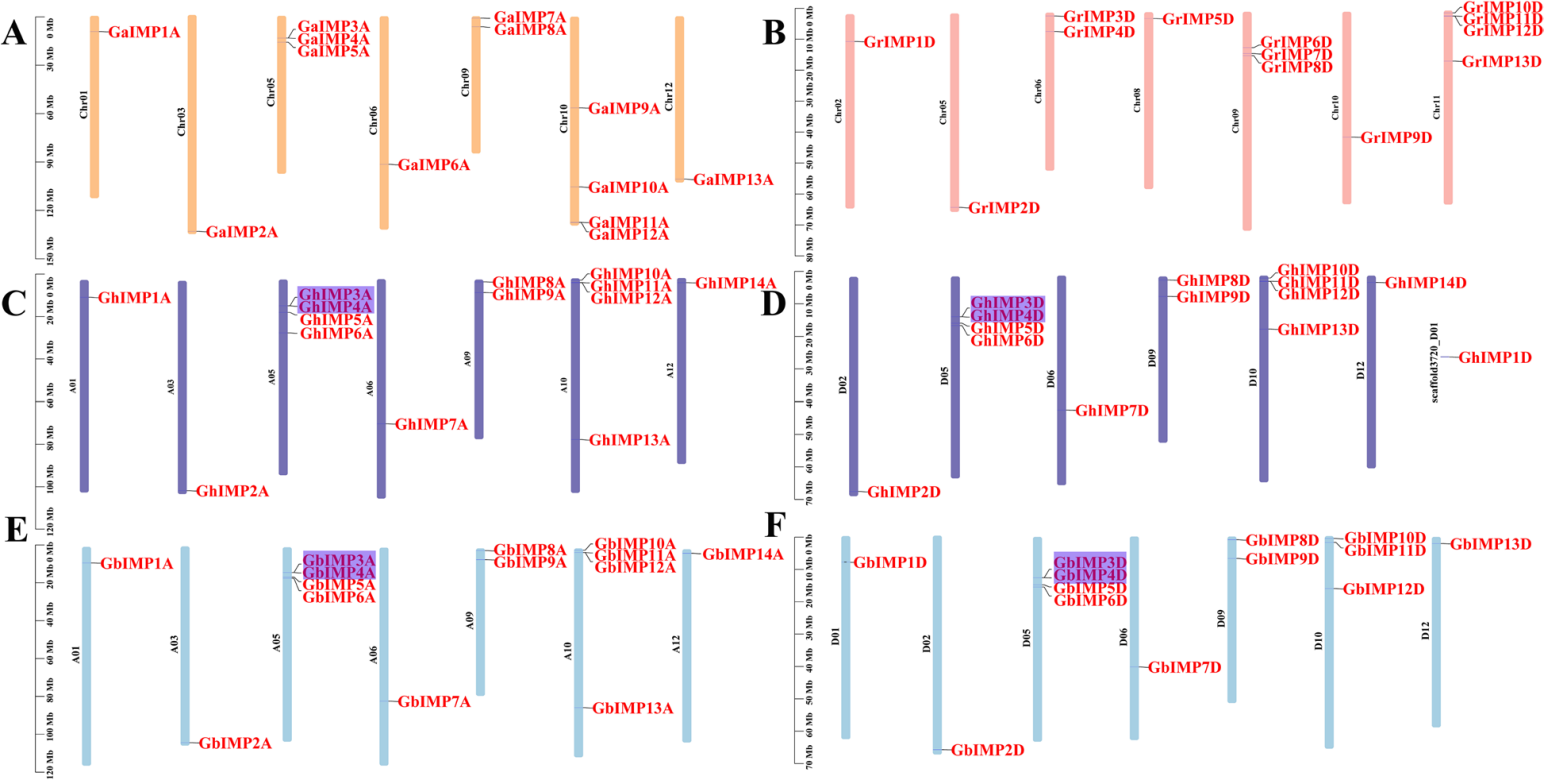
Chromosomal location of *IMP* genes from four *Gossypium* species. **A** Chromosome distribution of *IMP* genes in *G. arboretum*. **B** Chromosome distribution of *IMP* genes in *G. raimondii*. **C** Chromosome distribution of *IMP* genes in *G. hirsutum* At sub-genome (GhAt). **D** Chromosome distribution of *IMP* genes in *G. hirsutum* Dt sub-genome (GhDt). **E** Chromosome distribution of *IMP* genes in *G. barbadense* At sub-genome (GbAt). **F** Chromosome distribution of *IMP* genes in *G. barbadense* Dt sub-genome (GbDt). The gene ID on each chromosome corresponds to the approximate location of each *IMP* gene. The purple shades were identified as tandem duplicated gene pairs

To visualize the specific distribution of genes on chromosomes of four cotton species, we mapped the physical locations of these genes on cotton chromosomes by using TBtools software (Fig. 3). 148 *IMP* genes were unevenly located on different specific chromosomes, of which only *GhIMP1D* gene was mapped on scaffold.

Except for GhAt05-A05 and GbAt05-A05, the number of *IMP* genes on the chromosomes of the GhAt and GbAt subgenomes was the same as that of the diploid homologous chromosome A genome (*G. arboretum*) (Table 1), most of the genes in subgroup A were preserved during evolution, and only the genes on chromosome 05 were increased. These results showed that this may be due to segmental duplication or whole genome duplication events during evolution, and it could also be the inaccuracy of genome sequencing.

**Table 1:** Comparison of chromosomes harboring number of *IMP* genes from different genomes and subgenomes of four *Gossypium* species (Ga, Gr, Gh, Gb)

Chr. No	Ga	Gh-At	Gb-At	Gr	Gh-Dt	Gb-Dt
Chr.01	1	1	1	0	0	1
Chr.02	0	0	0	1	1	1
Chr.03	1	1	1	0	0	0
Chr.04	0	0	0	0	0	0
Chr.05	3	4	4	1	4	4
Chr.06	1	1	1	2	1	1
Chr.07	0	0	0	0	0	0
Chr.08	0	0	0	1	0	0
Chr.09	2	2	2	3	2	2
Chr.10	4	4	4	1	4	3
Chr.11	0	0	0	4	0	0
Chr.12	1	1	1	0	1	1
Chr.13	0	0	0	0	0	0
Scaffold	0	0	0	0	1	0
Total	13	14	14	13	14	13

However, compared with the A genome, the situations of GhDt/D and GbDt/D in D subgroup were completely different. Except for GhDt01/02/03/04/07/13–01/02/03/04/07/13 and GbDt02/03/04/07/13-A05, the number of *IMP* genes on the chromosomes of the GhDt and GbDt subgenomes was not same as that of the diploid homologous chromosome D genome (*G. raimondii*) (Table 1). Specifically, compared with *G. raimondii*, 3, 3 and 1 genes were increased on chromosome 05, 10 and 12 in *G. hirsutum*, respectively, and in *G. barbadense*, 3, 2 and 1 genes were increased on chromosome 05, 10 and 12, respectively. 1, 1, 1 and 4 genes were decreased on chromosome 06, 08, 09 and 11 in *G. hirsutum*, while 1, 1, 1 and 4 genes were decreased on chromosome 06, 08, 09 and 11 in *G. barbadense*, which may be related to the chromosome deletion of *G. hirsutum*/*G. barbadense* or the translocation of large fragments during the evolution.

### Gene duplication and collinearity analysis

**Fig. 4:**
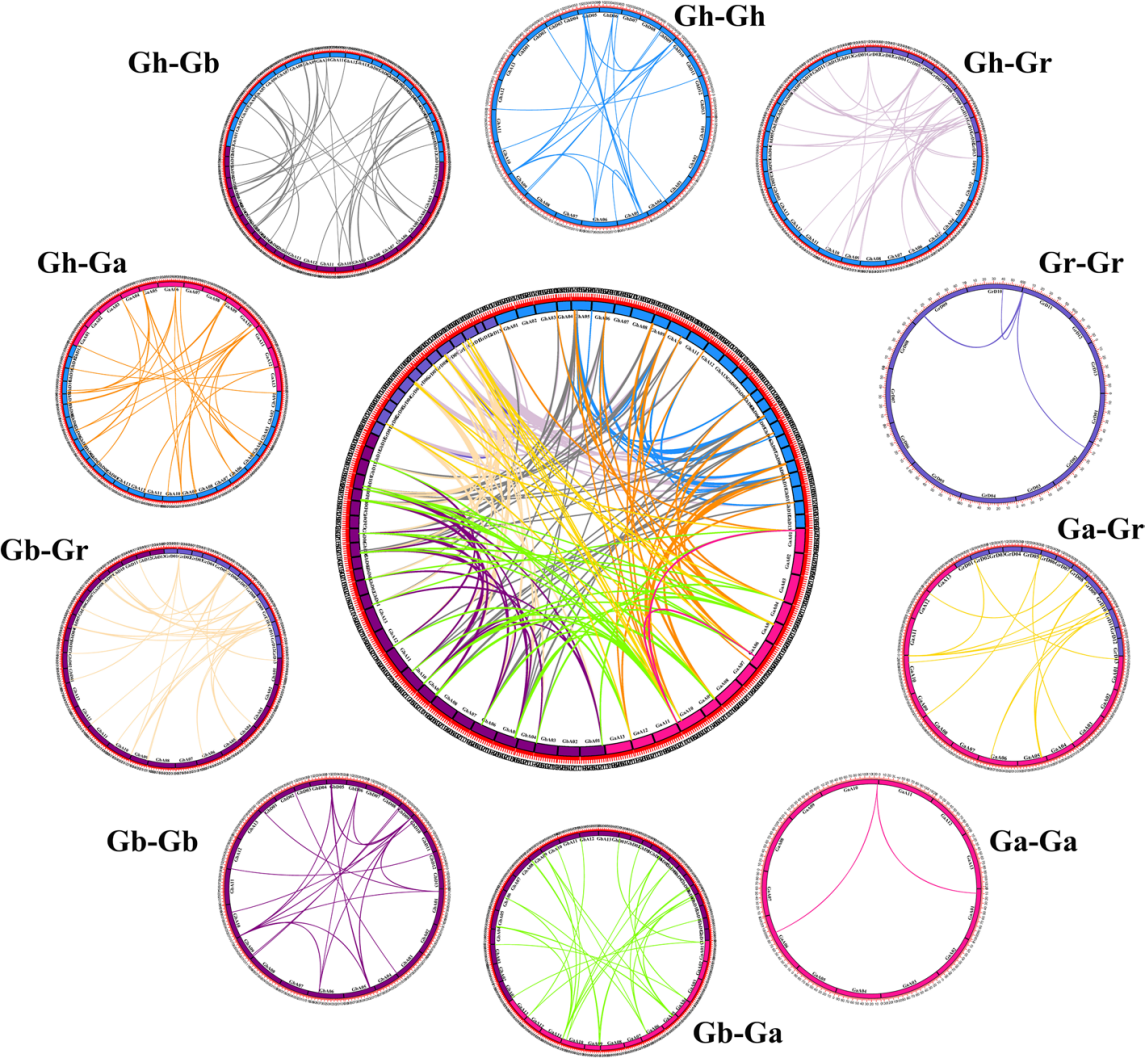
Syntenic relationship of duplicated *IMP* genes pairs from four *Gossypium* species. Lines with different color indicated different duplicated *IMP* gene pairs

Previous studies revealed that whole genome duplication, segmental duplication, and tandem duplication were the main drivers of plant gene family expansion [24]. To uncover the genome-wide duplicated principle of the *IMP* gene family in four *Gossypium* species, the duplicated gene pairs of four species were acquired and filtered by running MCScanX, four duplicated gene pairs (*GhIMP3A*/*4A*, *GhIMP3D*/*4D*, *GbIMP3A*/*4A* and *GbIMP3D*/*4D*) were identified as events of tandem duplication (Fig. 4), while the rest of duplicated gene pairs were detected as segmental duplication and whole genome duplication, indicating that compared to tandem duplication, the segmental duplication played a predominant role in the evolution of *IMP* gene family.

**Fig. 5:**
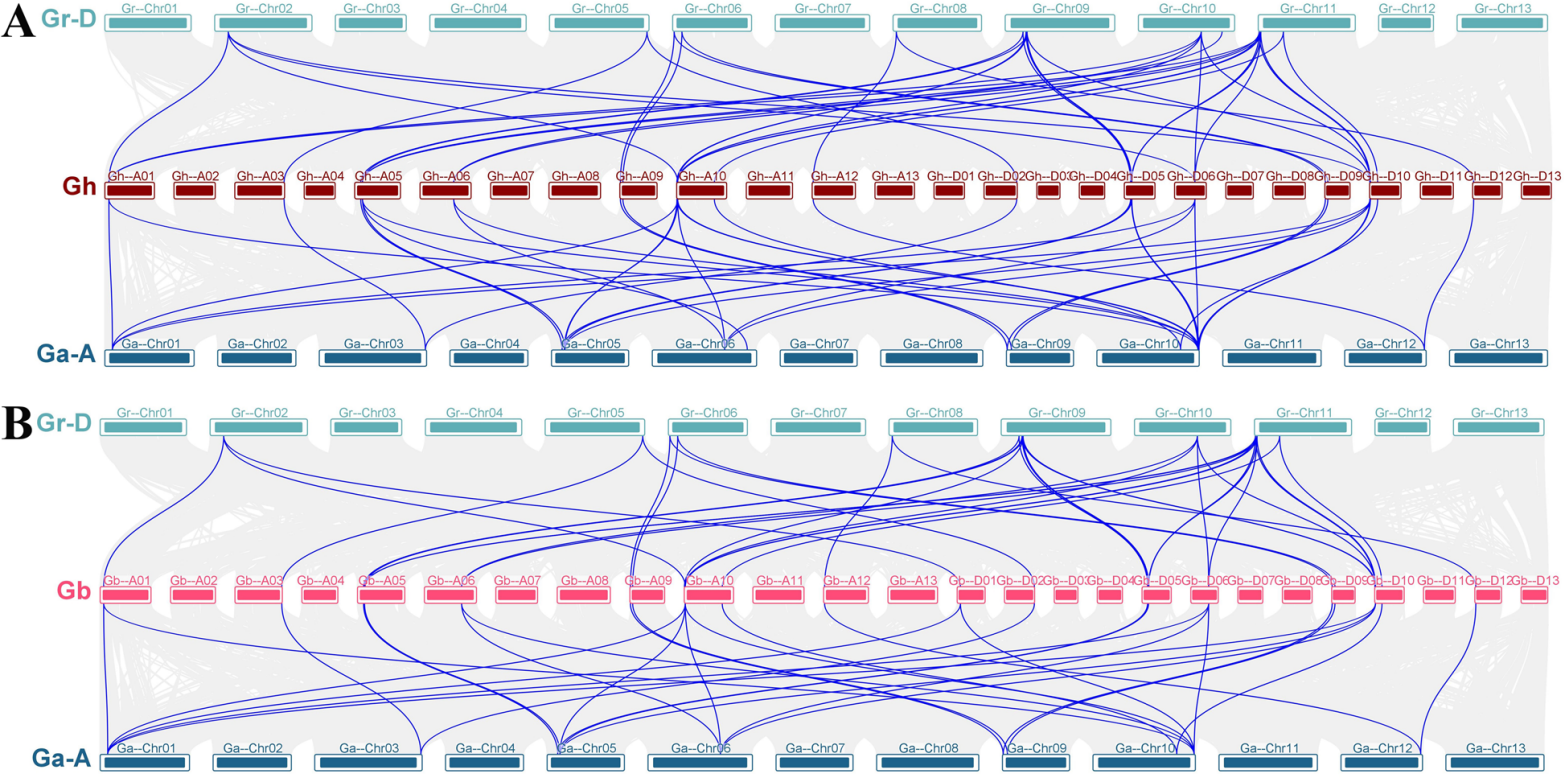
Synteny analyses of *IMP* genes between two allotetraploid cotton species and two diploid cotton species. Gray lines in the background indicated the collinear blocks among different genomes, blue lines indicated the syntenic *IMP* gene pairs

*G. hirsutum* and *G. barbadense* are allotetraploid species, produced by the hybridization between diploid A and D genome species [25]. To understand the evolutionary relationship between tetraploid and diploid, we further analyzed the collinear relationship among GhAt-A, GbAt-A, GhDt-D, GbDt-D (Fig. 5). There were 36, 38, 41, and 39 duplicated gene pairs in GhAt-A, GbAt-A, GhDt-D, GbDt-D, indicating that Gh and Gr had a closer relationship in the process of evolution.

### Analysis of *Ka*/*Ks*

**Fig. 6:**
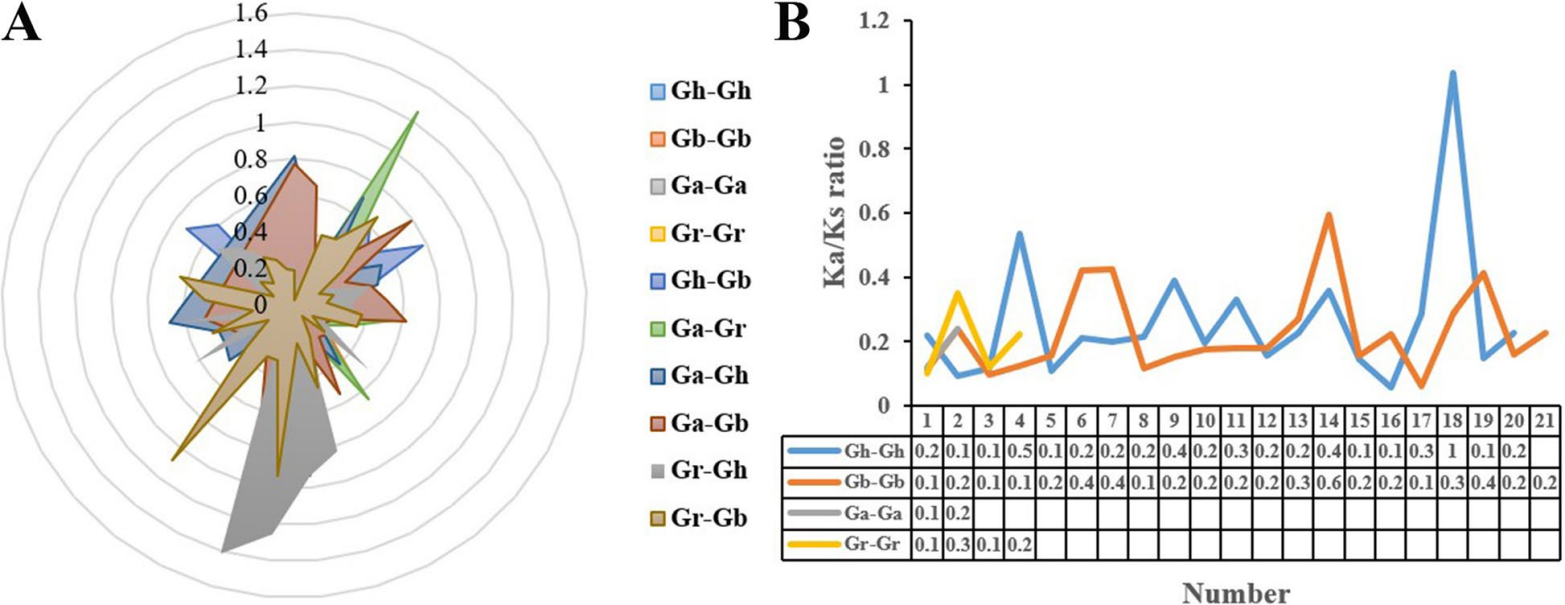
Schematic diagram of non-synonymous (*Ka*) to synonymous (*Ks*) ratio in cotton. **A** *Ka* and *Ks* divergence values in ten different pairs. **B** *Ka* and *Ks* divergence values in Gh-Gh, Gb-Gb, Ga-Ga, Gr–Gr

To further understand the selection pressure of the duplicated gene pairs in *IMP* gene family, we analyzed the *Ka*, *Ks*, and *Ka*/*Ks* ratio of 10 combined duplicate gene pairs (Gh-Gh, Gb-Gb, Ga-Ga, Gr-Gr, Gh-Gb, Ga-Gr, Ga-Gh, Ga-Gb, Gr-Gh, Gr-Gb). Among the 210 duplicated gene pairs, 5 (2.38%) pairs with *Ka*/*Ks* > 1, demonstrating that these genes may undergo the positive selection pressure. 205 (97.62%) pairs with *Ka*/*Ks* < 1 (172 pairs with 0 < *Ka*/*Ks* < 0.5, 33 pairs with 0.5 < *Ka*/*Ks* < 1), based on the *Ka*/*Ks* ratio, the *IMP* genes mainly underwent purifying selection (Fig. 6).

### Analysis of conserved protein motifs and gene structure

**Fig. 7:**
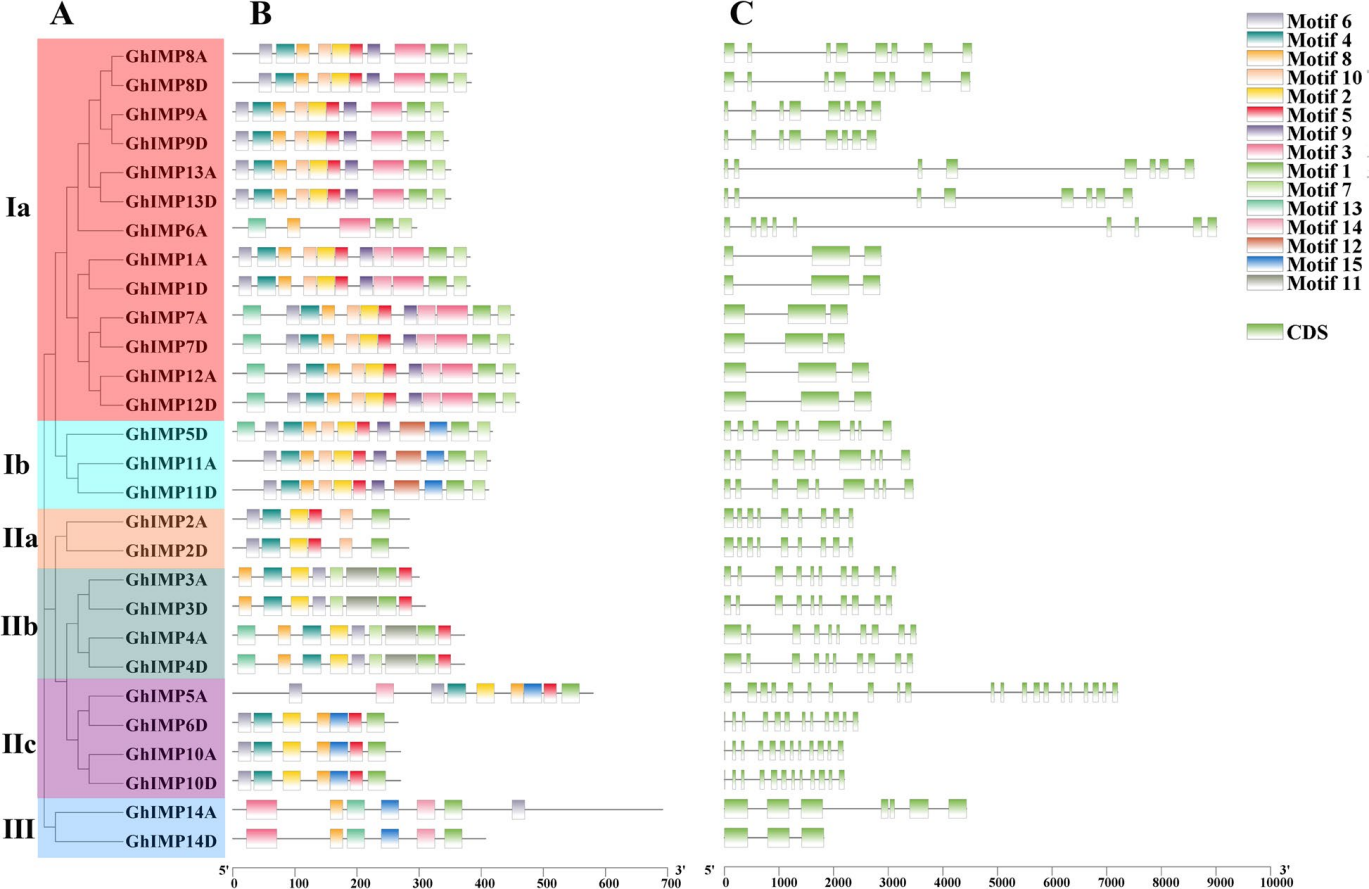
Analysis of phylogenetic tree, conserved protein motifs, and gene structure of *GhIMPs*. **A** Phylogenetic tree of *GhIMPs*. **B** Conserved motifs of *GhIMPs*. **C** Gene structure of *GhIMPs*. The green box represents the exon, and black lines indicate the introns

To explore the genetic structure characteristics of upland cotton, we analyzed the conserved motifs and gene structures of 28 *GhIMP* genes in *G. hirsutum* by using the online website MEME, and 15 motifs were selected. In general, according to the schematic diagram (Fig. 7), we can see that different clades of *GhIMP* genes have different types and numbers of motifs and gene structures. Except for *GhIMP6A* in subgroup Ia and *GhIMP6A* in subgroup IIc, the types and numbers of motifs in other subgroups were almost the same. The number of motifs was different in each clade, among them, clade I had the largest number of motifs, 10–12, and the number of motifs in subgroup IIa was the least, 6. Interestingly, some motifs were found only in certain subgroups. For example, Motif 15 was found only in some subgroups (Ib, IIC, and III), Motif 12 was found only in Ib, and Motif 5 was found in all subgroups except clade III.

In addition, we also analyzed the number of exons of 28 *GhIMP* genes (Fig. 7C, Supplementary Table S1), and the results showed that the number of exons varied from 3 to 21, and the largest number of exons was 21, which was *GhIMP5A*. The number of exons in the same subgroup was generally the same, which was well reflected in the subgroups Ib, IIa and IIb, which proved that these subgroups were relatively conserved in evolution. Gene characteristics, including the protein lengths, molecular weights (MWs), isoelectric points (pIs), grand average of hydropathy, and subcellular locations were showed in Supplementary Table S1. It was observed that the protein lengths varied from 266 amino acids (GhIMP6D) to 692 amino acids (GhIMP14A), and the average number of amino acids was 382. Among them, there were 7 genes with 200–300 amino acids, accounting for 25% of the total genes, and 11 genes with 300–400 amino acids, accounting for a large percentage (39%), there are 8 genes with 400–500 amino acids, accounting for 28% of the total, and 2 genes with more than 500 amino acids, accounting for 8% of the total genes. MWs ranged from 28.672 kDa (GhIMP6D) to 75.892 kDa (GhIMP14A), with an average MWs of 41.55, and the average pIs for *GhIMPs* was 6.36. Prediction of the subcellular localization of GhIMP proteins revealed that most proteins function in the chloroplast and cytoplasm, but subsequent experiments were needed to verify the location of proteins.

### Gene ontology (GO) annotation analysis of *GhIMP* genes

**Fig. 8:**
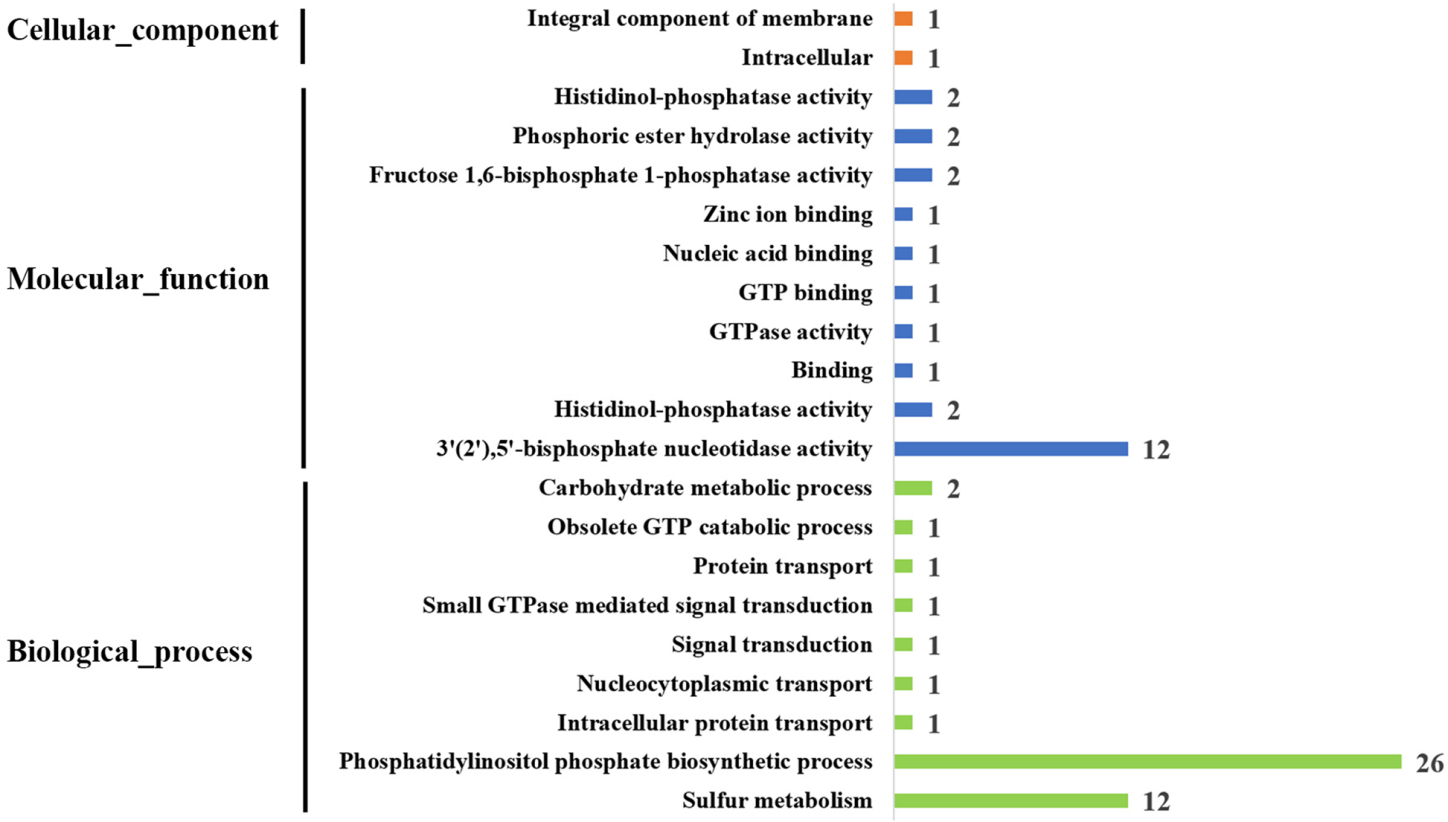
Gene ontology (GO) annotations of *GhIMP* genes

To study the function of *GhIMP* genes more intuitively, we used CottonFGD to analyze their GO annotations, which including cellular component, molecular function, and biological process. As shown in Fig. 8, *GhIMP* genes were categorized into 21 functional groups under main three categories. Phosphatidylinositol phosphate biosynthetic process (GO:0046854) was the most abundant biological process, with 26 genes. 3'(2'),5'-bisphosphate nucleotidase activity (GO:0008441) was the most enriched molecular function, with 12 genes.

### Analysis of promoters and differentially expressed genes

**Fig. 9:**
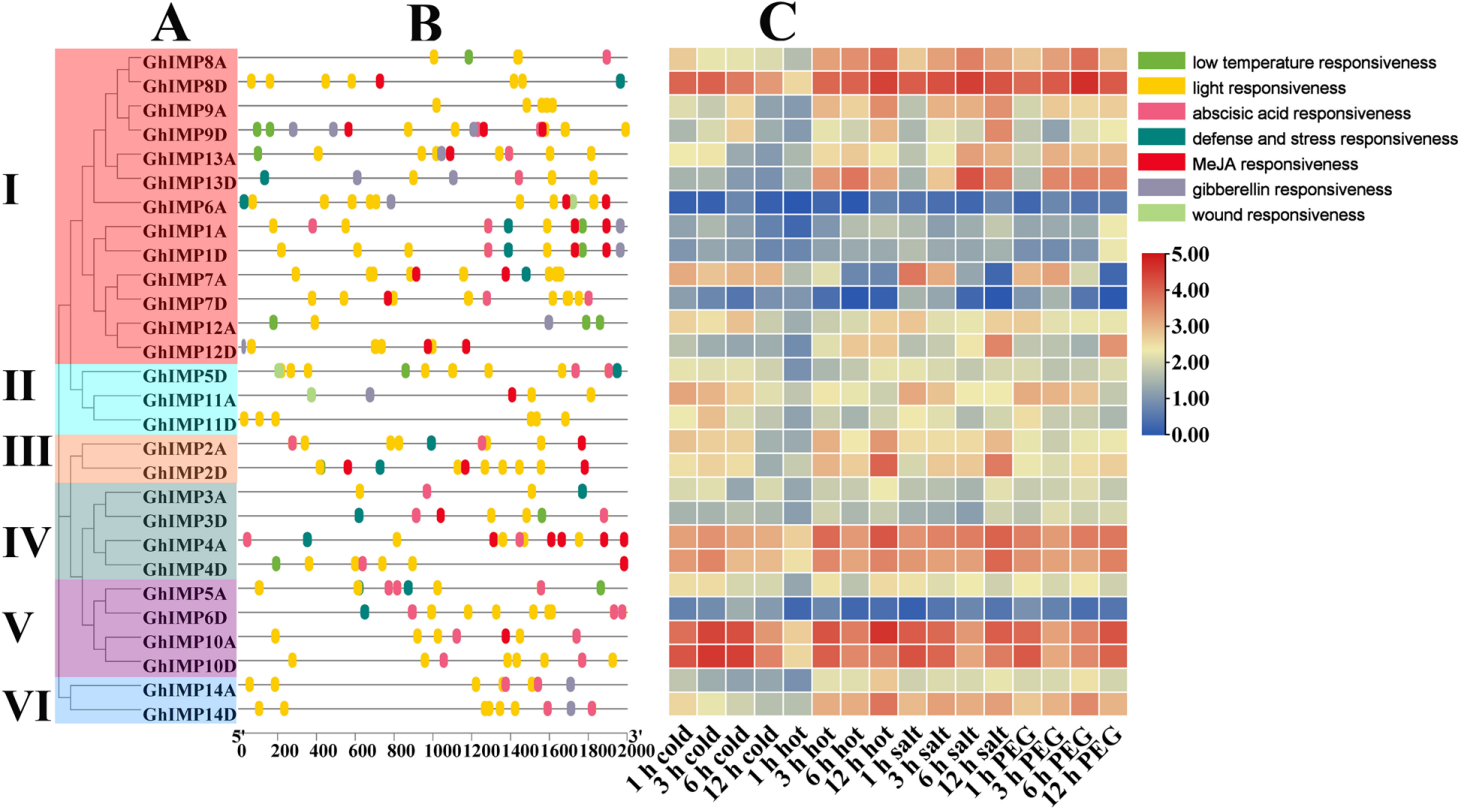
*Cis*-acting element analysis and differentially expressed genes (DEGs) analysis of *GhIMP* genes. **A** Phylogenetic tree of *GhIMPs*. **B** *Cis*-acting elements in promoters of *GhIMPs*. **C** Heatmap of *GhIMPs* under cold, hot, salt, and PEG stress. The relative expression of heat map was characterized by log_2_ transformed

Promoters can interact with transcription factors to precisely regulate transcription initiation and transcription efficiency of genes, and *cis*-acting elements located in the promoter region were found to play vital roles in the response to abiotic stress [26]. The promoters of *GhIMP* family genes were analyzed, and we found that most promoters contained hormone-related elements (abscisic acid response element, MeJA response element, gibberellin response element) and stress-related elements (low temperature response element, defense and stress response element, wound response element) (Fig. 9A, B). All genes had light-responsive elements, and each gene contained more than one light responsive element. For example, *GhIMP6A* contained 7 light responsive elements. 16 of 28 *GhIMP* genes contained MeJA responsive elements, accounting for 57% of the total, and 19 (67.86%) of 28 genes contained abscisic acid (ABA) responsive elements.

Then the expression patterns of *GhIMP* genes under different abiotic stress including cold, hot, salt and PEG stress were analyzed (Fig. 9C). In general, different genes have different expression patterns under different stresses. The expression levels of three genes increased significantly after various stress treatments, namely *GhIMP8D*, *GhIMP10D* and *GhIMP10A*, while 3 genes decreased significantly, namely *GhIMP7D*, *GhIMP6D* and *GhIMP6A*.

### qRT-PCR of *GhIMP* genes in response to NaHCO_3_ stress

**Fig. 10:**
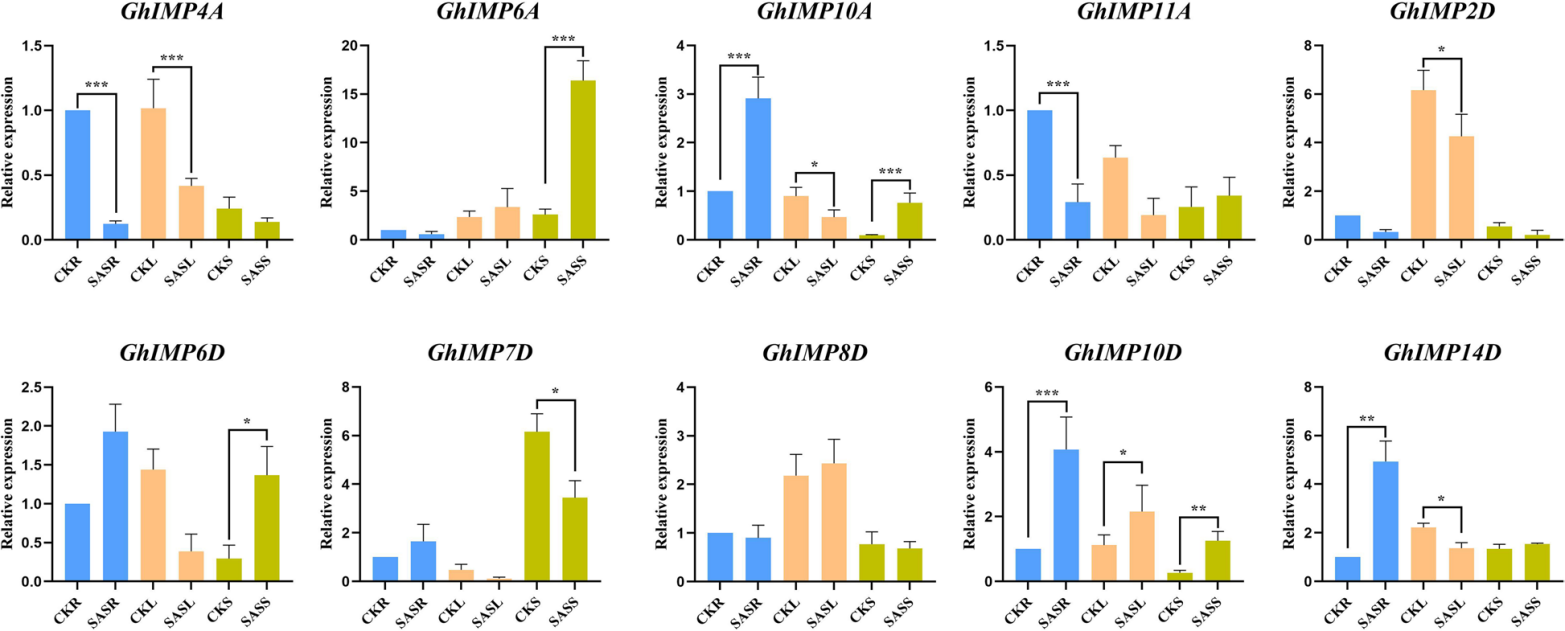
Expression analysis of *GhIMP* genes in response to SAS stress after 12 h in leaves, stems and roots using qRT-PCR assays. The mean values were from three independent biological replicates. Statistical analyses were performed by Student’s t-test (*P < 0.05, **P < 0.01 and *** *P* < 0.001). Notes: CK: ddH_2_O, SAS: 125 mM NaHCO_3_, R: Root, S: Stem, L: Leaf

To further confirm the response of *GhIMP* genes to alkaline stress, 10 *GhIMP* genes from different clades were randomly selected for qRT-PCR to detect their expression profiles in roots, stems and leaves treated with 125 mM NaHCO_3_ (Fig. 10). The treatment of the concentration of 125 mM NaHCO_3_ was carried out according to our previous research results [12]. The expression pattern of 5 genes was the same, namely *GhIMP4A*, *GhIMP6A*, *GhIMP2D*, *GhIMP8D* and *GhIMP10D*. The expressions of *GhIMP6A* and *GhIMP10D* genes increased under alkaline stress, and *GhIMP10D* significantly up-regulated in leaves. The remaining genes showed inconsistent expression profiles in roots, stems and leaves, suggesting that these genes may be involved in different pathways. In leaves, only the expression of *GhIMP10D* was significantly upregulated, and we speculated that this gene may play an important role in leaves, and we will conduct a preliminary study of its function in leaves.

### Exogenous ABA can promote the expression of *GhIMP* genes

**Fig. 11:**
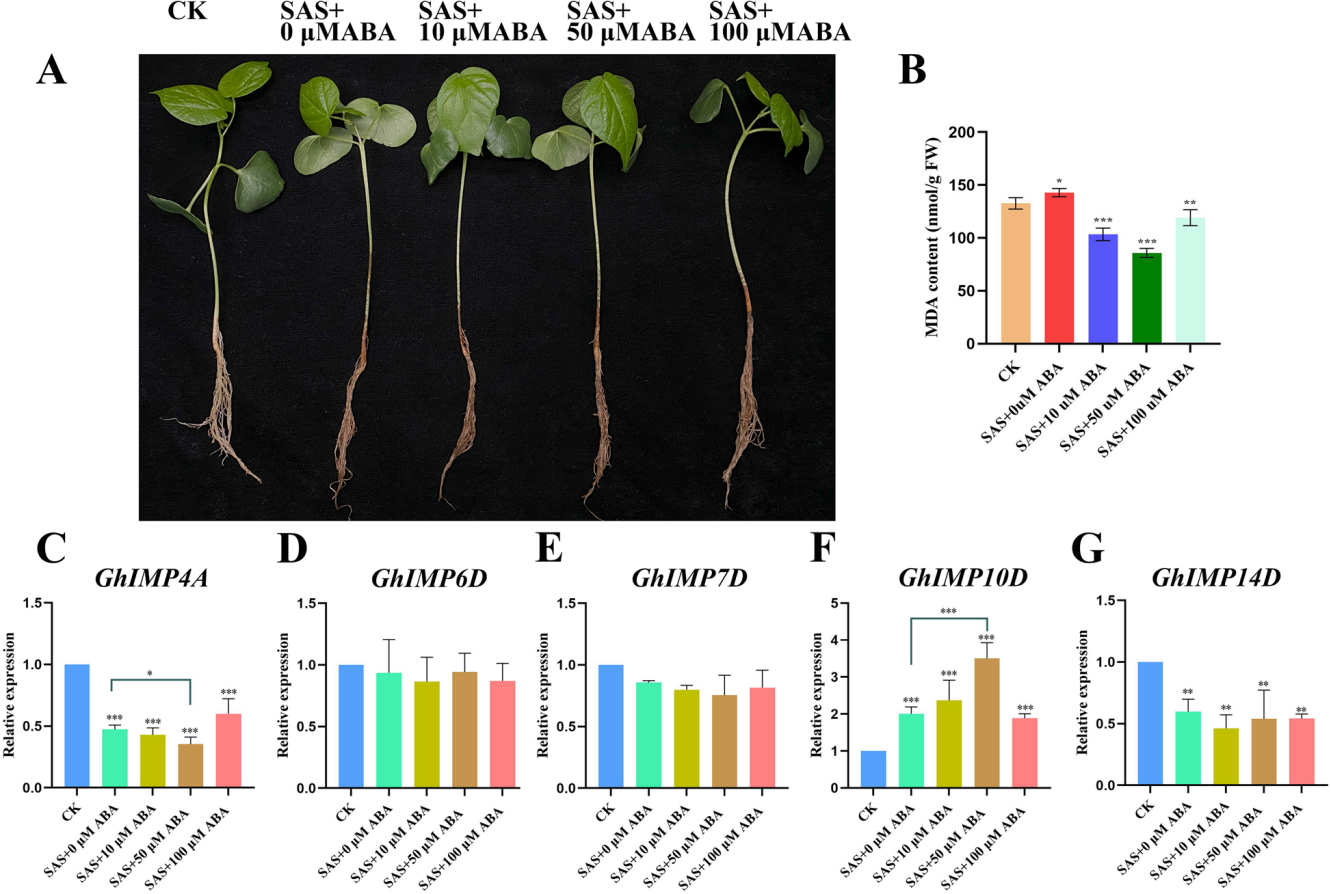
Alleviating effect of exogenous ABA on cotton seedings under alkaline stress and expression levels of *GhIMP* genes contained ABA *cis*-acting elements. **A** Phenotypes of cotton seedlings treated with different concentrations of ABA under alkaline stress. **B** MDA content of cotton seedlings treated with different concentrations of ABA under alkaline stress. **C**-**G** The relative expression levels of *GhIMP* genes contained ABA *cis*-acting elements. Notes: SAS: 125 mM NaHCO_3_

By analyzing the *cis*-acting elements of *GhIMP* genes (Fig. 9), we found that most *GhIMP* genes contained ABA responsive *cis*-acting elements. 2 of the genes contained 3 ABA *cis*-acting elements, namely *GhIMP6D* and *GhIMP5A*. 9 genes contained 2 ABA *cis*-acting elements, namely *GhIMP1A*, *GhIMP2A*, *GhIMP5A*, *GhIMP14A*, *GhIMP3D*, *GhIMP5D*, and *GhIMP14D*. To investigate the alleviating effect of ABA on alkaline stress and whether ABA can promote the expression of *GhIMP* genes, different concentrations of ABA (0, 10, 50 and 100 μM) were applied to cotton seedlings at three-leaf stage under NaHCO_3_ treatment (Fig. 11). The results showed that the exogenous application of 50 μM ABA could alleviate the damage of alkaline stress, at the same time, the MDA contents were measured, and compared with 0 μM ABA, the MDA content in cotton seedlings with 50 μM ABA was significantly reduced. In addition, we measured 5 genes containing more ABA *cis*-acting elements, including *GhIMP4A*, *GhIMP6D*, *GhIMP7D*, *GhIMP10D* and *GhIMP14D*, and found that only *GhIMP4A*, *GhIMP10D* and *GhIMP14D* genes were significantly induced by ABA, and the expression of the remaining two genes did not change significantly.

### Three-dimensional (3D) structure prediction of GhIMP proteins

**Fig. 12:**
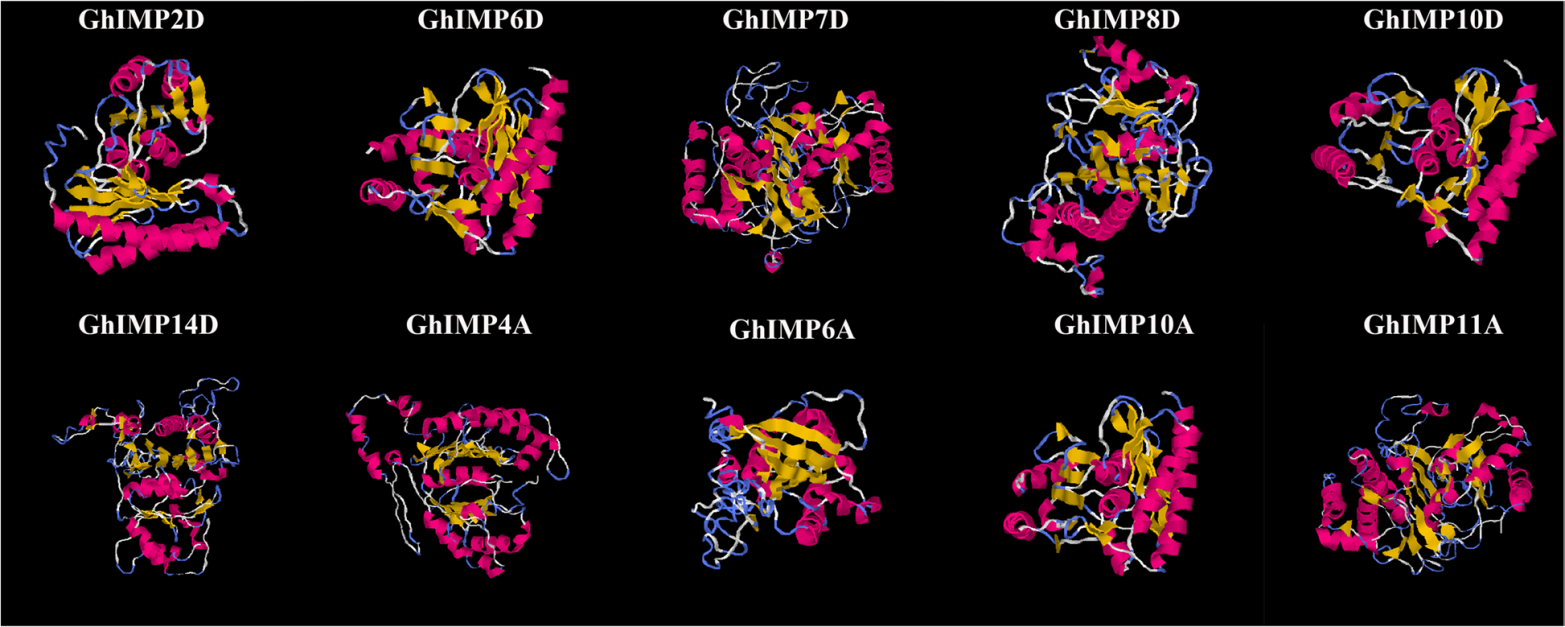
3D structure of 10 GhIMP proteins. α-helices are indicated by red, β-strands are indicated by yellow, and random coils are indicated by blue

To understand the protein structure of the GhIMP proteins, I-TASSER was used to obtained the 10 GhIMP proteins’ structures. The modeled structures for the selected GhIMP proteins had 8 (GhIMP2D, GhIMP6D, GhIMP10D and GhIMP10A) to 15 (GhIMP7D) α-helices, and 10 (GhIMP8D) to 14 (GhIMP14D) were identified as β-strands (Fig. 12). The same clade had similar protein structures, such as GhIMP10D, GhIMP6D, and GhIMP10A in clade IIc, which was different from the protein structure of GhIMP14D in clade III.

### Interaction network of GhIMP10D protein

**Fig. 13:**
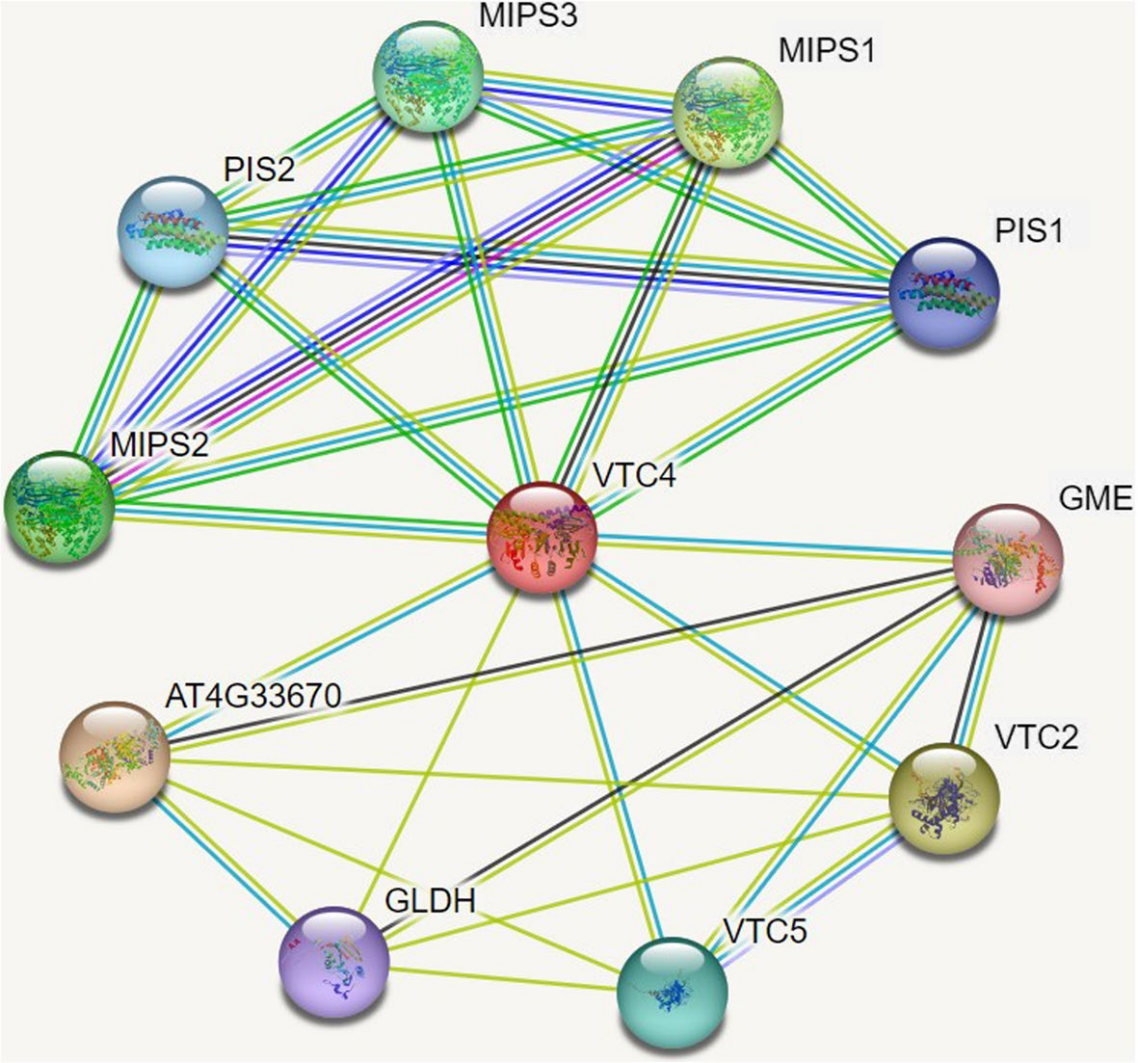
Protein interaction network of GhIMP10D. VTC4 is the corresponding name of GhIMP10D protein on String

To study the possible regulatory mechanism of GhIMP10D protein, we used the online website STRING to predict the possible interaction proteins of GhIMP10D based on VTC4, the homologous protein with the highest homology to GhIMP10D in *Arabidopsis* (Fig. 13). GhIMP10D mainly interacted with six types of proteins, including L-galactose 1,4-lactone dehydrogenase (GLDH), Inositol-3-phosphate synthase (MIPS1, MIPS2, and MIPS3), GDP-L-galactose phosphorylase (VTC2 and VTC5), GDP-D-mannose 3',5'-epimerase (GME), CDP-diacylglycerol-inositol 3-phosphatidyltransferase (PIS1 and PIS2) and NAD(P)-linked oxidoreductase (AT4G33670).

### Silencing *GhIMP10D* gene is sensitive to alkaline stress

**Fig. 14:**
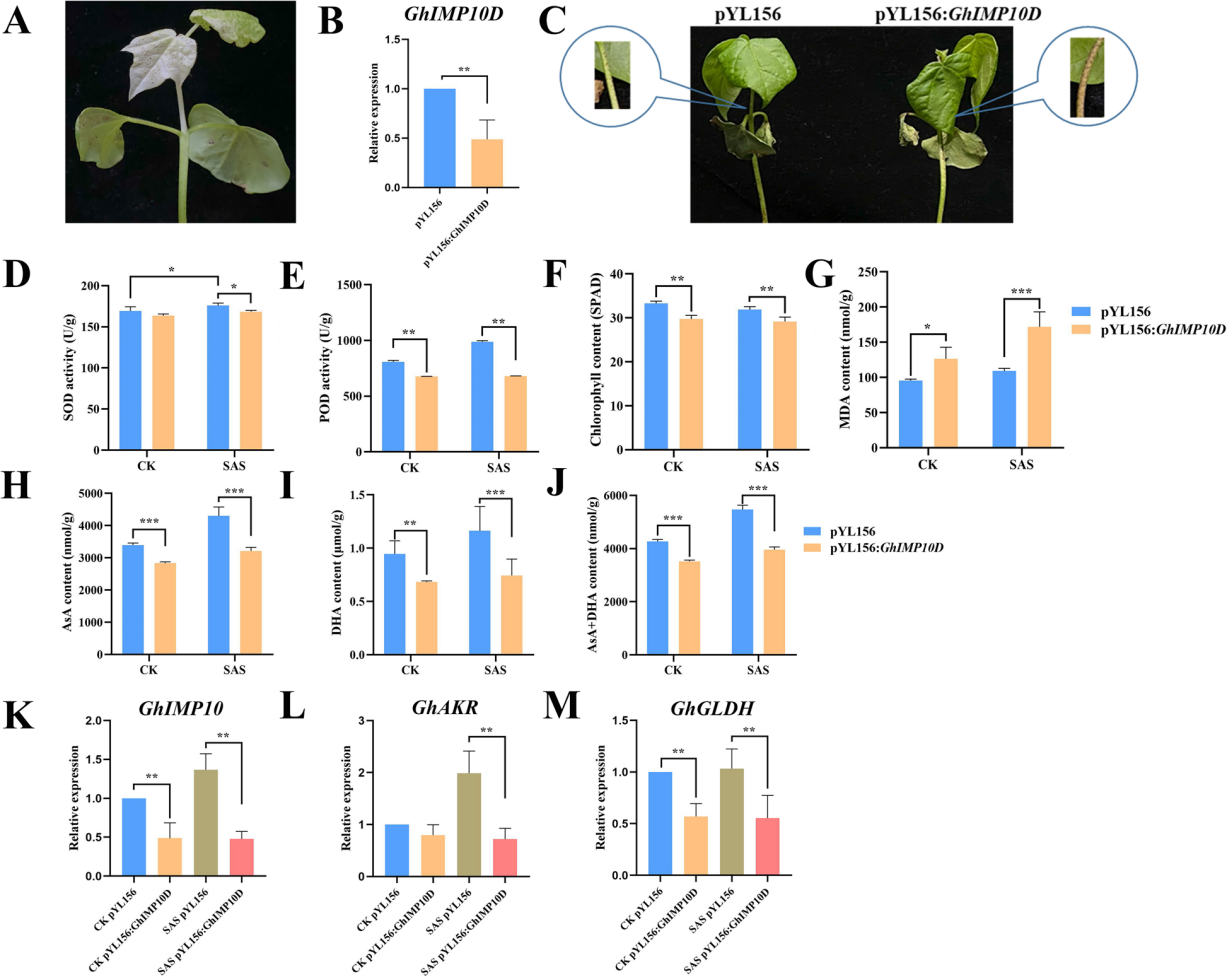
VIGS experiments of the *GhIMP10D* gene and measurement of physiological indicators. **A** The true leaves of pYL156: PDS cotton showed bleaching, which proved that the VIGS system was effective. **B** qRT-PCR analysis of pYL156 and pYL156: *GhIMP10D* plants*.* **C** Phenotypes of pYL156 and pYL156: *GhIMP10D* cotton seedlings under alkaline stress. **D-G** Physiological indicators including SOD, POD, chlorophyII and MDA of pYL156 and pYL156: *GhIMP10D* cotton seedlings under ddH_2_O and alkaline treatment. **H-J** AsA, DHA and total AsA contents of pYL156 and pYL156: *GhIMP10D* cotton seedlings under ddH_2_O and alkaline treatment. **K-M** Relative expression of genes associated with the AsA synthesis pathway. Notes: SAS: 125 mM NaHCO_3_

To further investigate the function of *GhIMP10D* gene under alkaline stress, VIGS were performed to preliminarily investigate the gene function. Firstly, qRT-PCR was performed on pYL156 and pYL156: *GhIMP10D* plants when the albino phenotype was present, and the expression level of pYL156: *GhIMP10D* plants was significantly decreased (Fig. 14). Then, cotton seedlings were subjected to 125 mM NaHCO_3_ alkaline stress treatment, and it was found that cotton seedlings with silenced *GhIMP10D* gene were sensitive to alkaline stress, indicating that *GhIMP10D* gene could positively regulate cotton alkaline tolerance. At the same time, we measured the content of ascorbic acid (AsA) and chlorophyII and found that the AsA and chlorophyII content of cotton seedlings silenced with the *GhIMP10D* gene was significantly lower than that of pYL156 plants. In addition, the activities of some antioxidant enzymes were also measured, and the results showed that compared with pYL56 plants, the content of SOD and POD under alkaline stress in silenced plants were decreased significantly, and the content of MDA was increased significantly. In summary, we suggested that *GhIMP10D* gene may positively regulate tolerance to NaHCO_3_ stress by regulating the activity of antioxidant enzymes and the content of AsA to eliminate ROS.

## Discussion

AsA is a kind of secondary metabolic substance that is beneficial to both plant and human health, and understanding the biosynthetic pathways of ascorbic acid and the role of key genes in the synthesis of AsA is of great significance for increasing the content of AsA in crops. At the same time, AsA has a strong ability to remove ROS, so it is of great significance to study the resistance of AsA to saline-alkaline stress.

Cotton is a standard model for studying the evolution of polyploids [27], so we used genomic data to explore the evolutionary relationship of cotton in four species. In this study, 28, 27,13 and 13 *IMP* genes was identified in *G. hirsutum*, *G. barbadense*, *G. arboreum*, and *G. raimondii*, respectively. In *G. hirsutum*, 14 *IMP* genes were identified in the At subgenome, and 14 *IMP* genes were identified in the Dt subgenome. The numbers of *IMP* genes in the *G. hirsutum* and *G. barbadense* were almost the sum of those in *G. arboreum* and *G. raimondii*, which suggesting that 2 allotetraploid *G. hirsutum* and *G. barbadense* species may resulted from hybridization of the two ancestral species [21].

The expansion of gene families caused by gene duplication events is one of the main evolutionary mechanisms leading to functional diversification and speciation [28]. To better understand the expansion mechanism of *IMP* gene family, intragenomic and intergenomic 10 duplication events (Ga-Ga, Ga-Gb, Ga-Gr, Gb-Gb, Gb-Gr, Gh-Ga, Gh-Gb, Gh-Gh, Gh-Gr and Gr-Gr) were analyzed by MCScanX (Fig. 4). Only *GhIMP3A*/*4A*, *GhIMP3D*/*4D*, *GbIMP3A*/*4A* and *GbIMP3D*/*4D* were identified as tandem duplicated gene pairs, but none of the other combinations, indicating that segmental duplication was the main driving force of *IMP* gene family evolution.

To illuminate the divergence after gene duplication, the *Ka*, *Ks* and *Ka*/*Ks* of duplicated gene pairs were calculated (Fig. 6A). In general, The *Ka*/*Ks* ratio could predict a gene's evolutionary background, and *Ka*/*Ks* < 1 represented that the gene had undergone purification selection, eliminating deleterious mutations and preserving important protein structures, *Ka*/*Ks* > 1 indicated that the gene had undergone positive selection, which accelerated the evolution of the gene, *Ka*/*Ks* = 1 was neutral selection, which indicated that natural selection does not lead to gene mutation [29]. In this study, more than 93% of the duplicated gene pairs with *Ka*/*Ks* values less than 1, indicating that the *IMP* gene family had undergone strong purification selection.

The increase or decrease in exon-introns can be caused by the integration and recombination of gene fragments. It was found that gene structure variation can cause the divergence of gene families in the process of evolution [24]. The genes in subgroup IIc had the largest number of exons, 12 (*GhIMP6D*, *GhIMP10D*, *GhIMP10A*) and 21 (*GhIMP5A*), respectively. Motif 5 was present in all the other subgroups, but disappeared in clade III. Combined with the phylogenetic tree, we speculated that the genes of clade III may lose Motif 5 during the evolution. The presence of Motif 6 in all genes proved that it preserved as a conserved motif in the process of evolution. Motif 12 was found only in Ib, which might indicate they had acquired important functions during evolution, and this might be used as a basis to identify this subgroup, similarly, in other gene family, there was a subfamily that had only one particular motif [30]. *GhIMP* genes of the same subfamily had similar gene structures and motif compositions (Fig. 7B, C), and high conservation of genes in the same subfamily suggested that the *GhIMP* genes had undergone duplication events during evolution. In summary, the differences in gene structure and motif composition among the different subfamilies suggested that their functions may be different.

Approximately 90% of AsA in cells was reported to be localized in the cytoplasm, and most *GhIMP* genes were predicted to be localized in the cytoplasm in our study. Therefore, we hypothesized that *GhIMP* genes may play an important role in the regulation of AsA content in the cytoplasm. The subcellular localization of *GhIMP* genes need to be further verified in the future.

*Cis*-acting elements play an important role when plants are subjected to abiotic stresses [31]. In our study, several stress-relevant *cis*-acting elements were identified, such as low temperature response elements, defense and stress response elements and wound response elements, which suggesting that *GhIMP* genes might play a critical role in response to abiotic stresses (Fig. 9B). All *GhIMP* family genes contained light-responsive *cis*-acting elements, proving that light may induce the expression of *GhIMP* family genes. Previous studies reported that the expression of *LeIMP1* in tomato increased with the exposure to light, indicating that the *IMP* gene responded positively to light [32]. In general, phytohormone signals will induce various adaptive responses that ultimately regulate plant physiological and biochemical processes in response to various abiotic stresses [33]. ABA plays a crucial role in combating abiotic stress, it was reported that ABA signaling pathway responded positively to alkaline stress. *GsSKP21* mediated the ABA signaling pathway to resist alkaline stress by changing the expression levels of ABA signaling related genes and ABA-induced genes in *Glycine soja* [34]. It was found that ABA could enhance alkaline stress tolerance in rice by upregulating genes related to antioxidant defense and stress tolerance [35]. In our study, most *GhIMP* genes had abscisic acid response elements, and combined with previous research, we speculated that the *GhIMP* genes may protect against alkaline stress by regulating the ABA signaling pathway, especially genes containing multiple abscisic acid response elements, such as *GhIMP3D*, *GhIMP5D*, *GhIMP7D*, *GhIMP10D*, *GhIMP2A*, *GhIMP5A*, *GhIMP10A* and *GhIMP14A*. In addition, MeJA-responsive *cis*-acting elements were also enriched in *GhIMP* family members, accounting for 57.14% of the total number, and almost all subgroups Ia and IIa contained this element. Moreover, several *GhIMP* genes also contained gibberellin responsive *cis*-acting elements (39.28%), most of which were concentrated in subgroups Ia and III. Previous studies showed that AsA content regulated the expression of plant defense genes and controlled plant growth and development through the hormone signaling pathway [36]. Therefore, *IMP* genes was likely to regulate hormone signaling pathway by regulating AsA synthesis in response to stress, and relevant experiments needed to be further verified.

ABA, as an important plant hormone, plays a vital role in response to various abiotic stresses, especially drought, salt and low temperature stress [37]. It was found that exogenous ABA can promote the expression of genes to resist the damage to various abiotic stresses. For example, in *Grapevine*, exogenous ABA can promote the expression of *VviZIP* gene and detoxification related genes to reduce the toxic effect caused by zinc accumulation [38], and it was found that exogenous ABA could induce the expression of galactinol synthase and raffinose synthase genes to increase tolerance to cold stress [39]. In *A. thaliana*, ABA could upregulate the expression of *ZAT6*, thus positively regulates Cd accumulation and tolerance [40]. In *Oryza sativa*, ABA could upregulate *OsSMP1*, and overexpression of *OsSMP1* gene could improve the tolerance to CdCl_2_ and CuSO_4_ [41]. In *Nitraria tangutorum*, exogenous ABA could significantly increase the transcription levels of *NtFLS*, *NtF3H*, *NtF3H* and *NtANR* genes participated in the synthesis pathway of flavonoid metabolites, while flavonoids were reported to improve tolerance to alkaline stress [42]. In Jute, *ABF* family genes *CoABF3* and *CoABF7* were significantly up-regulated under salt and drought stresses after exogenous application of ABA [43]. In this study, after adding different concentrations of ABA to cotton seedlings under alkaline stress, we found that exogenous ABA had a resistance to alkaline stress in cotton seedlings, which was consistent with previous studies. 5 *GhIMP* genes with 2 or 3 ABA *cis*-acting elements were detected by qRT-PCR, and the expression of the 2 genes was found to be unchanged after exogenous ABA treatment, namely *GhIMP6D* and *GhIMP7D*. The expression of the 3 genes changed significantly, among which *GhIMP4A* and *GhIMP14D* were significantly downregulated after ABA treatment, and *GhIMP10D* was significantly upregulated. suggesting that ABA treatment could induce the expression of *IMP* family genes in response to alkaline stress. Combined with the phylogenetic analysis, we found that the expression patterns of *IMP* family genes treated with ABA was different, and the genes in clade II and III may respond positively to ABA treatment. In conclusion, ABA can increase the expression of some *GhIMP* genes in response to alkaline stress, which is consistent with previous studies that *CaIMP* gene was induced under salinity, drought and heat stress and ABA treatment in *chickpea* [16].

Any two proteins that are jointly involved in a specific cellular process are considered functionally related, therefore, co-expressed proteins are modulated by the same transcriptional programs and share same or similar pathways [44]. In this study, we analyzed the interaction network of GhIMP10D protein. It was found that GhIMP10D protein mainly interacted with three types of proteins, namely GDP-L-galactose phosphorylase (GLDH), GDP-L-galactose phosphorylase (VTC2 and VTC5) and Inositol-3-phosphate synthase (MIPS). Previous studies reported that the GLDH and VTC played a vital role in the ascorbic acid synthesis pathway [45], and MIPS was an important enzyme in the phospholipid synthesis pathway [46]. GhIMP10D protein participated in the synthesis of AsA, and it also participated in the synthesis of inositol, which was consistent with previous studies that the enzyme encoded by IMP was a bimolecular enzyme [14, 47], indicating that GhIMP10D protein in cotton may also be a bimolecular protein. The specific proof needed to be verified by subsequent experiments.

Previous studies had found that *IMP* gene was induced by various stresses, the expression level of *CaIMP* was found to be increased in salt, cold, heat and dehydration stress with maximum expression with dehydration stress [16]. Transcript analyses showed that Rice *OsIMP* gene was significantly upregulated by cold and ABA treatment [15]. Similarly, *IMP* genes in *G. hirsutum* were induced by cold, hot, salt and PEG stresses, but the response of the *GhIMP* genes to varioust stresses was different. For example, *GhIMP6A*, *GhIMP6D* and *GhIMP7D* were significantly down-regulated compared with other genes. The expression levels of *GhIMP8D*, *GhIMP10A* and *GhIMP10D* under the four kinds of stresses were higher than that of other genes. Combined with the qRT-PCR data, we could know that the expression of *GhIMP10D* in leaves was the highest, so we speculated that it may play an important role in abiotic stress.

qRT-PCR results showed that *GhIMP10D* gene was strongly induced by alkaline stress and was differentially expressed in roots, stems and leaves, with the highest expression in leaves. Therefore, further analysis of this key gene is necessary. Previous studies showed that *IMP* genes were not only involved in seed development [48], AsA synthesis [14, 49], but also in response to various abiotic stresses [15, 50]. However, there is no relevant research of *IMP* genes under alkaline stress.

It was found that alkaline stress was more harmful to the photosynthetic system of cotton compared with salt stress [51]. In this study, we found that the chlorophyII content of both pYL156 and pYL156: *GhIMP10D* plants decreased significantly after alkaline stress, and the chlorophyII content of pYL156: *GhIMP10D* plants decreased significantly compared with pYL156, which was consistent with previous study. Alkaline stress could induce the production of ROS, and AsA was produced to eliminate excess ROS in the body. When the *GhIMP10D* gene was silenced, the content of total AsA decreased significantly. Therefore, we believed that *GhIMP10D* gene may regulate the response to alkaline stress by regulating the synthesis of AsA. To further verify our conjectures, we analyzed the expression levels of genes in the AsA synthesis pathway, and found that the expression levels of *GhAKR* and *GhGLDH* genes decreased significantly after *GhIMP10D* gene was silenced. Therefore, we believed that *GhIMP10D* gene regulates the response to alkaline stress by regulating the synthesis of AsA. In addition, we measured the activity of antioxidant enzymes to explore the influence of *GhIMP10D* gene on antioxidant enzymes. The results showed that the activities of SOD and POD decreased significantly after the silencing of *GhIMP10D* gene. Therefore, *GhIMP10D* gene can resist alkaline stress by regulating the activity of antioxidant enzymes.

**Fig. 15:**
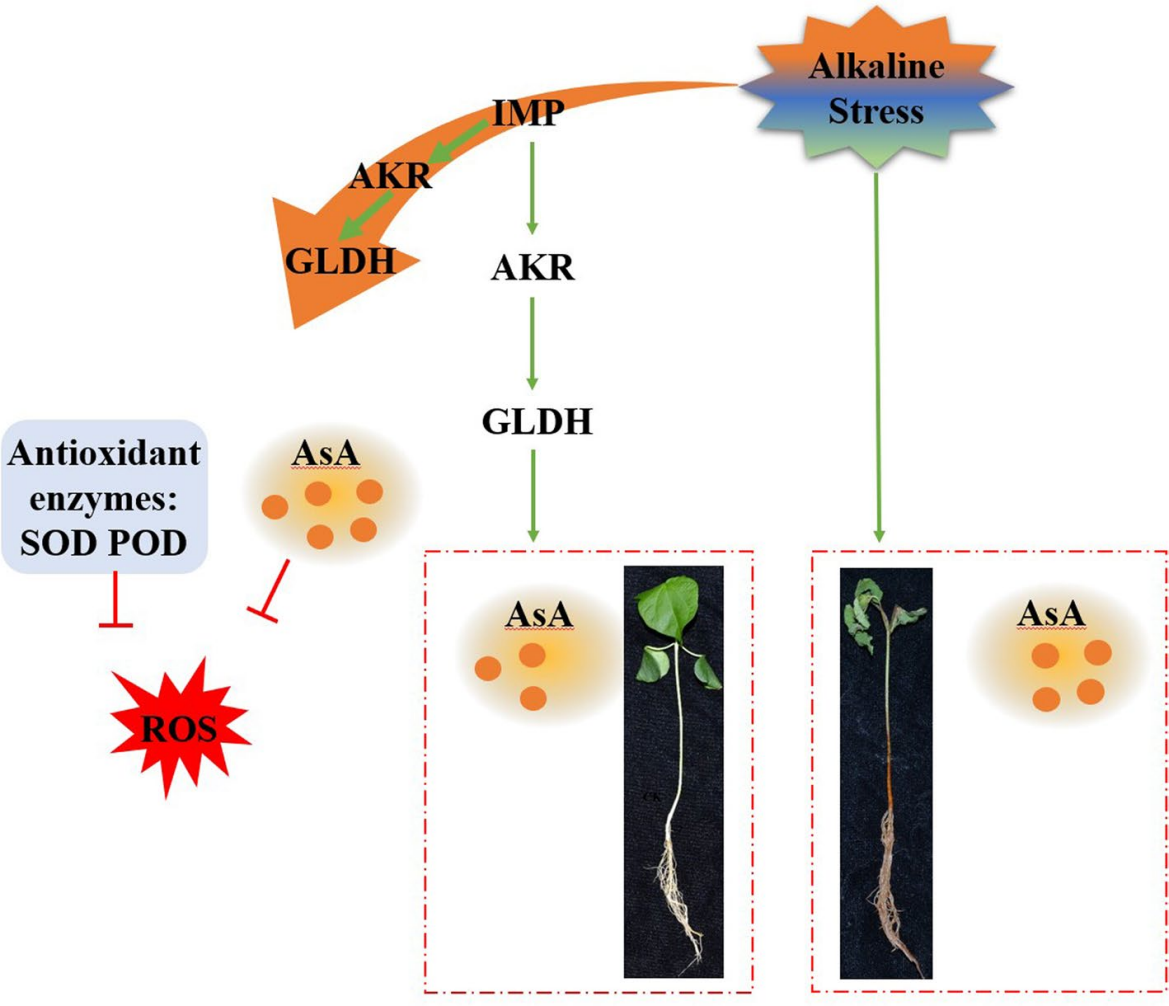
A working model of the function of *GhIMP10D* modulates AsA and antioxidant enzyme to eliminate ROS under alkaline stress

In summary, an alkaline-responsive gene, *GhIMP10D*, was identified as playing an important role in resisting alkaline stress in cotton, and *GhIMP10D* might respond to alkaline stress by controlling AsA synthesis through a complex regulatory mechanism (Fig. 15). Under normal circumstances, the redox state in cotton seedlings is balanced, and *GhIMP10D* controls the expression of *GhAKR* and *GhGLDH* genes to keep AsA contents stable. However, when seedlings are subjected to alkaline stress, a large amount of ROS is induced in *vivo*, in which *GhIMP10D* is induced to be expressed to resist alkaline stress. At the same time, the balance of ascorbic acid in the body is also broken, and the synthesis of ascorbic acid is increased to cope with alkali stress. In addition, *GhIMP10D* will resist alkaline stress through two ways, firstly, *GhIMP10D* will control the synthesis of antioxidants to eliminate excessive ROS in vivo, on the other hand, *GhIMP10D* will control the expression of *GhAKR* and *GhGLDH* genes to produce a large amount of AsA to eliminate excessive ROS in the body. The results reveal the function of *GhIMP10D* gene in the regulation of alkaline tolerance.

## Conclusions

With the deterioration of the environment, it is necessary to enhance the alkali-tolerant capability of cotton. Based on morphophysiological and bioinformatics analysis, the present study revealed that *GhIMP* genes had positive responses to various abiotic stresses, and *cis*-acting elements analysis of *GhIMP* genes showed that many *GhIMP* genes had elements associated with plant hormone and abiotic stress, of which ABA responsive elements accounted for a large proportion. Exogenous ABA was added under alkaline stress, and the results showed that ABA could induce the expression of some *GhIMP* genes. qRT-PCR results of *GhIMP* genes under alkaline stress showed that *GhIMP10D* was highly expressed in response to alkaline stress and ABA treatment. Silencing *GhIMP10D* gene in cotton made it sensitive to alkaline stress. Enhanced tolerance to alkaline stress might be because of shared role in regulating the expression of downstream *GhAKR* and *GhGLDH* genes to control the synthesis of AsA to remove ROS, in increasing the activity of antioxidant enzymes such as SOD, POD and chlorophyII content to eliminate ROS.

## Materials and methods

### Sequence retrieval and analysis

Genome sequences of four *Gossypium* species including *G. hirsutum*, NAU; *G. barbadense*, HAU; *G. arboreum*, CRI; and *G. raimondii*, JGI were used to identify the gene family. The genome sequence and annotation information of four species were downloaded from Cotton Functional Genomic Database (CottonFGD) (http://www.cottonfgd.org/) [52]. The IMP proteins of other 7 species (*Arabidopsis thaliana TAIR10*, *Glycine max Wm82.a4.v1*, *Populus trichocarpa v4.1*, *Theobroma cacao v2.1*, *Oryza sativa v7.0*, *Zea mays PHJ40 v1.2* and *Vitis vinifera v2.1*) were retrieved from Phytozome v13 (https://phytozome-next.jgi.doe.gov/) by using PF00459 as the keyword. The Hidden Markov models (HMMs) (version 3.0) profiles of PF00459 were downloaded from online website Pfam (https://pfam.xfam.org/) to obtain the IMP proteins with default parameters, then we further screened the IMP proteins by using NCBI Batch Web CD-Search Tool (https://www.ncbi.nlm.nih.gov/Structure/bwrpsb/bwrpsb.cgi) [53] and manually deleted individual family members with incomplete C and N terminals.

Finally, the acquired CDS sequences of *G. hirsutum* were submitted to CottonFGD to get the physicochemical parameters like protein length, molecular weight (MWs), isoelectric points (pIs) and grand average of hydropathy. Subcellular location of GhIMP proteins were predicted by using several online websites, such as WOLF-PSORT (https://wolfpsort.hgc.jp/) and CELLO v.2.5 (http://cello.life.nctu.edu.tw/) [54].

### Phylogenetic analysis and sequences alignments

Multiple sequence alignment of all these IMP proteins were performed using ClustalX program in MEGA7 software. Subsequently, MEGA 7 software was used to construct a phylogenetic tree of four *Gossypium* species using neighbor joining (NJ) method with default parameters. A phylogenetic tree of IMP proteins in the four *Gossypium* species and other 7 species was constructed using the Maximum Likelihood method of MEGA7 with 1000 bootstrap replicates based on the LG + G model.

### Chromosomal mapping of *IMP* genes from four *Gossypium* species

*IMP* genes of four *Gossypium* species were mapped on chromosomes using TBtools software with Generic Feature Format (gff) files and gene IDs downloaded from CottonFGD [55].

### Analysis of the conserved protein motifs and gene structure

Multiple Em for Motif Elicitation (MEME) website (http://meme-suite.org/) was used to identify the conserved motifs of GhIMP proteins, and the parameters used in this study were set as follows: maximum number of different motifs is 15, and other parameters are default [56]. The diagram of evolutionary relationship, gene structure, and conserved motifs of GhIMP proteins was drawn using TBtools software.

### Collinearity analysis of *IMP* genes in four *Gossypium* species

To investigate the collinearity relationship among *IMP* genes of four *Gossypium* species, we analyzed the duplicated gene pairs from four cotton species *G. hirsutum*, *G. arboreum*, *G. raimondii*, and *G. barbadense*, then complete genome sequences of four *Gossypium* species along with genome annotation files were subjected to MCScanX software to investigate the collinearity synteny relationship [57]. Finally, the diagrams were visualized using Advance Circos tool in TBtools software with chromosome length files and the genome alignment files among four *Gossypium* species.

### Calculation of selection pressure

To assess the selection pressure of duplicated gene pairs, the nonsynonymous (*Ka*), the synonymous (*Ks*), and *Ka*/*Ks* were calculated by using *Ka*/*Ks* calculator in TBtools software.

### Analysis of *cis*‑acting element in promoters of *GhIMPs*

The 2000 bp DNA sequence of the upstream region of *GhIMP* genes were obtained from the online website CottonFGD (https://www.cottonfgd.org/) as the promoters. The Plant CARE website (http://bioinformatics.psb.ugent.be/webtools/plantcare/html/) was used to predict the *cis*-acting elements in the promoter region of *GhIMP* genes. Finally, we constructed a schematic diagram of phylogenetic tree and *cis*-acting elements in TBtools software.

### Gene ontology analysis

CottonFGD was used to study the gene ontology annotations of *GhIMP* genes, and the annotations were based mainly on three aspects: biological process, cellular component, and molecular function.

### qRT-PCR analysis under NaHCO_3_ stress

To determine the response of *GhIMP* genes to alkaline stress, a total of 10 genes were randomly selected from each subfamily, and the relative expression levels of these 10 genes were determined by taking root, stem and leaf tissues after 125 mM NaHCO_3_ treatment for 12 h. The total RNA of roots, stems and leaves were extracted using EASYspin Plus Plant RNA Kit (Aidlab, Beijing, China). The cDNA was reverse using HiScript III RT SuperMix for qPCR (+ gDNA wiper) (Vazyme, Nanjing, China). Then qRT-PCR was performed using the Applied Biosystems@7500 Fast instrument with TransStart Top Green qPCR SuperMix (TransGen Biotech, Beijing, China). All operations were carried out in accordance with the manufacturer's instructions. The Actin gene was used as a control. 2^−△△CT^ method was used to calculate the fold change for each sample [58].

### Three-dimensional structure analysis of GhIMP proteins

To identify the protein structures of GhIMPs, I-TASSER [59] (https://zhanglab.ccmb.med.umich.edu/I-TASSER/) was used to analyze the protein structures (α-helices, β-strands and random coils) of 10 GhIMP proteins selected by qRT-PCR.

### Expression patterns under different stresses

To understand the expression pattern of *GhIMP* genes, we analyzed it using RNA-seq data (PRJNA490626) downloaded from NCBI website (https://www.ncbi.nlm.nih.gov/), which mainly included the expression levels under salt, PEG, cold, and heat stresses [60]. In addition, we analyzed the expression pattern of *GhIMP* genes under alkaline stress based on published RNA-seq data (GSE165472) [12].

### ABA application of cotton seedlings under alkaline stress

When the cotton seedlings reached three true leaves, exogenous ABA treatment was applied to cotton seedlings to observe the alleviating effect of alkaline stress. Upland cotton material Zhong 9807 was used for further experiments, and it was obtained from Cotton Research Institute, Chinese Academy of Agricultural Sciences with the accession ID: xcy2399. ABA (Solarbio) was dissolved in a small amount of absolute ethanol and then diluted with deionized water to the different concentrations (0, 10, 50 and 100 μM). The leaves of cotton were completely soaked by spraying with different concentrations of ABA, and then dark for 12 h under alkaline stress. Cotton leaves of each treatment were sampled for the measurement of MDA and qRT-PCR at 0 h and 12 h, respectively.

### Gene interaction network of GhIMP10D protein

The GhIMP10D protein interaction network was analyzed by the STRING database (https://string-db.org/) [44], and *Arabidopsis thaliana* orthologs was used to predict the interaction of GhIMP10D with other proteins in cotton.

### VIGS (Virus-induced gene silencing) experiment of *GhIMP10D* gene

The pYL156 vector was digested with *BamH I* and *Sma I* restriction enzymes, amplifying the 300 bp fragment specific to the *GhIMP10D* gene, ligated using ClonExpress II One Step Cloning Kit from Vazyme. After that, the constructed vector was transferred into *A. tumefaciens* LBA4404. Upland cotton material Zhong 9807 was used for further experiments, and it was obtained from Cotton Research Institute, Chinese Academy of Agricultural Sciences. The plant material was deposited at the gene bank of the Institute of Cotton Research, Chinese Academy of Agricultural Sciences, with the accession ID: xcy2399. The cultures, cells and the procedure of infection were handled as previously described [61]. When the albino phenotype appeared, qRT-PCR experiments and physiological indicators were measured.

### Measurement of ascorbic acid (AsA) and Dehydroascorbic acid (DHA) content

AsA and DHA content was determined using Ascorbic Acid (AsA) Content Assay Kit (BC1230) and Dehydroascorbic Acid (DHA) Content Assay Kit (BC1240) from Solarbio. 0.1 g cotton leaves were taken and AsA content was measured according to the manufacturer's procedure, and three biological replicates were taken for each sample.

### Measurement of chlorophyll, malondialdehyde (MDA) contents, peroxidase (POD) and superoxide dismutase (SOD) activity assay

The SPAD-502 PLUS measuring instrument (Konica Minolta (China) Investment Ltd) was used to detect the chlorophyl II contents in the leaves, and the same part of each leaf was taken for measurement. 0.1 g cotton leaves were taken to determine MDA, POD and SOD contents using kits from Nanjing Jiancheng Bioengineering Institute (A003-3–1, A084-3–1 and A001-3–2).

### Supplementary Information

**Additional file 1: Supplementary Table S1.** Gene locus ID and their proposed names of all observed species and the gene characteristics in G. hirsutum. **Supplementary Table S2.** Duplicated gene pairs in 10 combinations (Ga-Ga, Ga-Gb, Ga-Gr, Gb-Gb, Gb-Gr, Gh-Gh, Gh-Ga, Gh-Gb, Gh-Gr and Gr-Gr). **Supplementary Table S3.** Non-synonymous (Ka) and synonymous (Ks) divergence values for Ga-Ga, Ga-Gb, Ga-Gr, Gb-Gb, Gb-Gr, Gh-Gh, Gh-Ga, Gh-Gb, Gh-Gr and Gr-Gr. **Supplementary Table S4. **Primer pairs used for this experiment.

## Data Availability

All data supporting the conclusions of this article are provided within the article and its additional files. The genomics sequences are available in the CottonFGD (https://cottonfgd.org/), RNA-Seq data downloaded from NCBI (https://www.ncbi.nlm.nih.gov/) with accession number (PRJNA490626).
