# Supplementary material for: GhIMP10D, an inositol monophosphates family gene, enhances ascorbic acid and antioxidant enzyme activities to confer alkaline tolerance in Gossypium hirsutum L
Source: BMC Plant Biol. 2023 Sep 22;23:447. doi: 10.1186/s12870-023-04462-x (PMC10515029; doi:10.1186/s12870-023-04462-x)
Supplement: Supplementary file 1 — Additional file 1: Supplementary Table S1. Gene locus ID and their proposed names of all observed species and the gene characteristics in G. hirsutum. Supplementary Table S2. Duplicated gene pairs in 10 combinations (Ga-Ga, Ga-Gb, Ga-Gr, Gb-Gb, Gb-Gr, Gh-Gh, Gh-Ga, Gh-Gb, Gh-Gr and Gr-Gr). Supplementary Table S3. Non-synonymous (Ka) and synonymous (Ks) divergence values for Ga-Ga, Ga-Gb, Ga-Gr, Gb-Gb, Gb-Gr, Gh-Gh, Gh-Ga, Gh-Gb, Gh-Gr and Gr-Gr. Supplementary Table S4. Primer pairs used for this experiment. [file 12870_2023_4462_MOESM1_ESM.zip › Supplementary Table S4.docx]

**Supplementary Table S4.** Primer pairs used for this experiment.

| Gene ID | Primer pairs for this experiment (5'-3') | |
| --- | --- | --- |
| *Actin* | ATCCTCCGTCTTGACCTTG | TGTCCGTCAGGCAACTCAT |
| *qGhIMP4A* | CGGATAAGGTGGAGCAGTCT | TCTGCAACTCCCAGAGCTAC |
| *qGhIMP6A* | CTACGGACTATGTGCCGACT | TGGCCTTGCTGATGAGAGAA |
| *qGhIMP10A* | TGGCTGACAATGGTTCTCCT | GGATTATCTCGCCGGCTTTC |
| *qGhIMP11A* | ATCTGATGGGCGGATACTGG | ACAAGAGCCTGGACTCCAAA |
| *qGhIMP2D* | CTTGGCTTCCGGTTATGTGG | AGCTTGTTGCACGTGAAGTT |
| *qGhIMP6D* | GAAAGCCGGCGAGATAATCC | GCCTCAGTGACCAAATCGAC |
| *qGhIMP7D* | ACTTGGCCACCTTGATGGTA | GCGGTGATGATACGTTAGCC |
| *qGhIMP8D* | TGTGCTGGCTTGTCCAAATC | GAACCATCAAGTGGCTGCAT |
| *qGhIMP10D* | GAAAGCCGGCGAGATAATCC | CACAAGCAGCAGTGGTTTCT |
| *qGhIMP14D* | TAGCTGCTGGCTATGTCCTC | AGACCTTCAGGCCAATCGAA |
| *V-GhIMP10D* | GCCTCCATGGGGATCC CCTATAAGAGTTTCATCTCA | CGAGACGCGTGAGCTCATCAAAATCTTTCCCGAACG |
